# Resilience and charge-dependent fibrillation of functional amyloid: Interactions of *Pseudomonas* biofilm-associated FapB and FapC amyloids

**DOI:** 10.1016/j.jbc.2024.108096

**Published:** 2024-12-18

**Authors:** Nimrod Golan, Amit Parizat, Orly Tabachnikov, Eilon Barnea, William P. Olsen, Daniel E. Otzen, Meytal Landau

**Affiliations:** 1 Department of Biology, Technion-Israel Institute of Technology, Haifa, Israel; 2 Interdisciplinary Nanoscience Center (iNANO), Aarhus University, Aarhus C, Denmark; 3 CSSB Centre for Structural Systems Biology, Deutsches Elektronen-Synchrotron DESY, Hamburg, Germany; 4 The Center for Experimental Medicine, Universitätsklinikum Hamburg-Eppendorf (UKE), Hamburg, Germany; 5 European Molecular Biology Laboratory (EMBL), Hamburg, Germany

**Keywords:** amyloid, Faps, biofilm, self-assembly, Pseudomonas, fibrillation, protein stability

## Abstract

FapC and FapB are biofilm-associated amyloids involved in the virulence of *Pseudomonas* and other bacteria. We herein demonstrate their exceptional thermal and chemical resilience, suggesting that their biofilm structures might withstand standard sterilization, thereby contributing to the persistence of *Pseudomonas aeruginosa* infections. Our findings also underscore the impact of environmental factors on functional amyloid in *Pseudomonas* (Fap) proteins, suggesting that orthologs in different *Pseudomonas* strains adapt to specific environments and roles. Challenging previous assumptions about a simple nucleation role for FapB in promoting FapC aggregation, the study shows a significant influence of FapC on FapB aggregation. The interaction between these FapB and FapC is intricate: FapB stabilizes FapC fibrils, while FapC slows down FapB fibrillation but can still serve as a cross-seeding template. This complex interplay is the key to understanding their roles in bacterial biofilms. Furthermore, the study highlights distinct differences between Fap and *Escherichia coli*'s CsgA (curli) amyloid, where CsgB assumes a simple unidirectional role in nucleating CsgA fibrillation, emphasizing the importance of a comprehensive understanding of various amyloid systems. This knowledge is vital for developing effective intervention strategies against bacterial infections and leveraging the unique properties of these amyloids in technological applications such as novel bionanomaterials or protective coatings.

The opportunistic pathogen *Pseudomonas aeruginosa* is a significant cause of mortality in patients with co-morbidities, particularly those with cystic fibrosis (CF) and ventilator-associated pneumonia (1, 2, 3, 4, 5). The resistance of this bacterium to various antibiotics underscores the urgent need for novel treatment approaches (1, 4, 5, 6, 7). In CF patients, chronic respiratory infections caused by *P. aeruginosa* typically begin in adolescence and can persist for decades (2, 3). The bacterium adapts to the pulmonary environment by forming biofilms that adhere to the respiratory epithelium, thereby evading the immune response and shedding immunogenic structures like pili and flagella (1, 2, 3).

Biofilms, which enhance surface adherence and create a protective environment for bacterial growth, are observed in *Pseudomonas* strains across diverse environments (8, 9, 10). In 2010, Dueholm *et al.* provided evidence of amyloid fibril involvement in *Pseudomonas* biofilm formation (11). Amyloids are known for their remarkable stability, resistance to protease digestion and denaturation, and self-polymerizing capabilities, serving as robust structural elements (4, 12, 13, 14). In microbes, amyloids can serve as scaffolds in biofilm matrices, especially under harsh and energy-depleted conditions (4, 8, 9, 10, 15). Amyloid fibrils may also act as surfactants and adhesins and are thought to play a role in bacterial quorum sensing systems (16, 17).

Bacterial amyloids are regulated by specific pathways, beneficial for the physiology of the organism, frequently aided by an assortment of accessory proteins (18). In *Pseudomonas*, amyloid production is orchestrated by the six-gene operon named functional amyloid in *Pseudomonas* (Fap), with FapC and FapB serving as the amyloid-forming subunits (4, 8, 11, 19). In enterobacteria, the curli system is tightly controlled by two operons, CsgBAC and CsgDEFG, with CsgA as the primary amyloid subunit and CsgB as a nucleator of CsgA fibrillation (20). Drawing parallels with the curli system, it was hypothesized that Faps serve similar roles as CsgA/B (8, 9, 10, 11, 21, 22, 23). Accordingly, FapB could function as a nucleator, accelerating the rapid elongation of FapC fibrils on the cell surface, or it might be integral to the structure of the mature fibril, thereby altering its physicochemical characteristics (4, 11, 19, 20). FapA influences the distribution of FapC and FapB in mature fibrils, with its deletion leading to FapB-dominated biofilm fibrils (8, 9, 11, 21, 24). FapD, a putative cysteine protease, may initiate secretion before degradation (25), while FapE's role as an extracellular chaperone is still being explored. The membrane protein FapF forms trimeric β-barrel channels, as observed by a crystal structure (25), crucial for extracellular Fap component secretion (4, 8, 24, 25). Fap proteins display various virulence traits. Removing FapC in *P. aeruginosa* reduces virulence, as shown in *Caenorhabditis elegans* models and polymorphonuclear neutrophil leukocytes phagocytosis assays (26, 27). The Fap system is not exclusive to *P. aeruginosa*; other pathogenic bacteria also possess Fap-encoding genes, like *Aeromonas caviae*, and *Laribacter hongkongensis*, known to cause gastroenteritis and diarrhea, as well as *Burkholderia gladioli*, *B. pseudomallei*, *Ralstonia pikettii*, and *Stenotrophomonas maltophilia*, which are associated with airway and lung infections (26, 28, 29, 30, 31).

Advances in structural biology have shed light on the stability and complex nature of eukaryotic amyloids. The prominent structural feature of amyloids is the formation of cross-β fibrils comprising paired β-sheets made of β-strands aligned perpendicularly to the fibril growth axis (4, 13, 32, 33). In contrast to human amyloids, understanding bacterial amyloids has been hampered by a lack of high-resolution structural data. A breakthrough came with the first crystal structure of bacterial amyloid, the cytotoxic PSMα3 from *Staphylococcus aureus*, revealing a polymorphic amyloid fold where α-helices can replace β-strands to form cross-α fibrils, contributing to its pathogenicity (34). Recent advancements in cryo-EM have begun to unveil the structures of biofilm-associated amyloids, highlighting both similarities and distinctions when compared to other amyloids found in humans and microbes. The cryo-EM structure of TasA from *Bacillus subtilis* consists of a fiber formed from folded monomers. These monomers are assembled through a donor–strand exchange mechanism, where each unit contributes a β-strand to complete the fold of the subsequent subunit in the fiber (35). In contrast, the structure of *Escherichia coli* curli CsgA, elucidated using a synergistic approach of cryo-EM and computational modeling, exhibited a unique globular β-solenoid configuration, composed of monomeric units that enable a quasi-homotypic stacking of β-strands (36). The predicted structures of FapC, FapB, and FapE modeled using AlphaFold (37, 38) suggest a β-solenoid which resembles the structure of CsgA (39, 40). The structural insights into Faps, CsgA, and TasA not only challenge traditional views of protein polymers and amyloid fibrils but also suggest that our understanding of functional amyloids may need reevaluation and expansion to encompass novel structural paradigms.

In this study, we investigate the molecular interactions between histidine-tagged FapB and FapC, the major amyloid-forming proteins of *P. aeruginosa*, specifically focusing on their fibrillation behavior and interaction dynamics. In addition, our research examines their fibrillation kinetics, sensitivity to pH changes and salt concentration, charge distribution, and their remarkable thermostability and chemical resistance. Contrary to the traditional view that FapB acts as a nucleator for FapC, our findings suggest that FapB does not serve this role in the PAO1 strain. Instead, FapC appears to have a more pronounced influence on FapB, affecting its fibrillation kinetic and leading to the formation of colocalized aggregates. Additionally, our results demonstrate the extreme stability of FapB and FapC fibrils, which are resistant to high temperatures and harsh chemical treatments such as formic acid (FA). This resilience highlights the significant challenges these amyloids pose in clinical and industrial settings, where standard sterilization methods may be insufficient to eradicate biofilm-associated infections. By elucidating the complex interplay between histidine-tagged FapB and FapC, our study provides new insights into the role of bacterial amyloids in biofilm stability and underscores the need for innovative approaches to combat *P. aeruginosa* biofilms.

## Results

### Effects of N- and C-terminal histidine tags on FapB and FapC fibrillation

In our study, we expressed and purified histidine-tagged FapB and FapC from the *P. aeruginosa* PAO1 strain (UniProt IDs Q9I2E9 and Q9I2F0, respectively) and assessed their fibrillation kinetics using a thioflavin T (ThT) fluorescence assay. While the histidine-tag is necessary for achieving high protein purity, the conditions required for its removal initiates rapid fibrillation of the Fap proteins which prevent assessment of fibrillation kinetics without the tag.

Fibrillation kinetics analysis using ThT fluorescence showed that 50 μM FapC with histidine tags, whether at the N-terminus or C-terminus, display similar kinetics across pH levels 5, 7, and 9 (Fig. S1). Since the C-terminal histidine-tag was associated with a slightly faster fibrillation rate, we chose this configuration for further testing. For FapB, the results differ: fibrillation occurs only when the tag is positioned at the N-terminus (Fig. S2), and we thus continued using this configuration for subsequent experiments. To elucidate the impact of the tag location on FapB, we employed AlphaFold (37, 38)-generated models of FapB monomer at varying pH levels, both with and without the tag at either end. The models revealed a folded monomer structure characterized by a β-solenoid with β-sheets that form interfaces akin to cross-β fibril structures (Fig. S2*B*). The experimental examination of a C-terminal histidine tag, featuring a short linker, demonstrated its interaction with FapB's surface as per the model. This interaction alters the charge distribution across different pH levels, potentially explaining the linker's role in inhibiting fibrillation (Fig. S2*A*). Conversely, the model suggests that the N-terminal tag, possessing a longer linker, does not directly interact with FapB's surface, correlating with the observed fibrillation capability of FapB tagged at the N-terminus.

### Differential sensitivity of FapB and FapC to pH and ionic strength conditions

*Pseudomonas* species thrive in diverse environments which vary significantly in pH, temperature, and salinity (4). We thus explored the differential sensitivity of histidine-tagged FapB and FapC to variations in protein concentrations, pH, and ionic strength. To study the influence of varying pH conditions on FapB and FapC fibrillation without changing the buffer, we used a “universal buffer,” which effectively controls pH from approximately 3.5 to 9.2 (41). Composed of 20 mM Tris–HCl, Bis-Tris, and sodium acetate, this buffer minimizes interference from metal ions like calcium and magnesium, ensuring precise pH control and enhancing the reliability of our experimental results.

**Figure 1:**
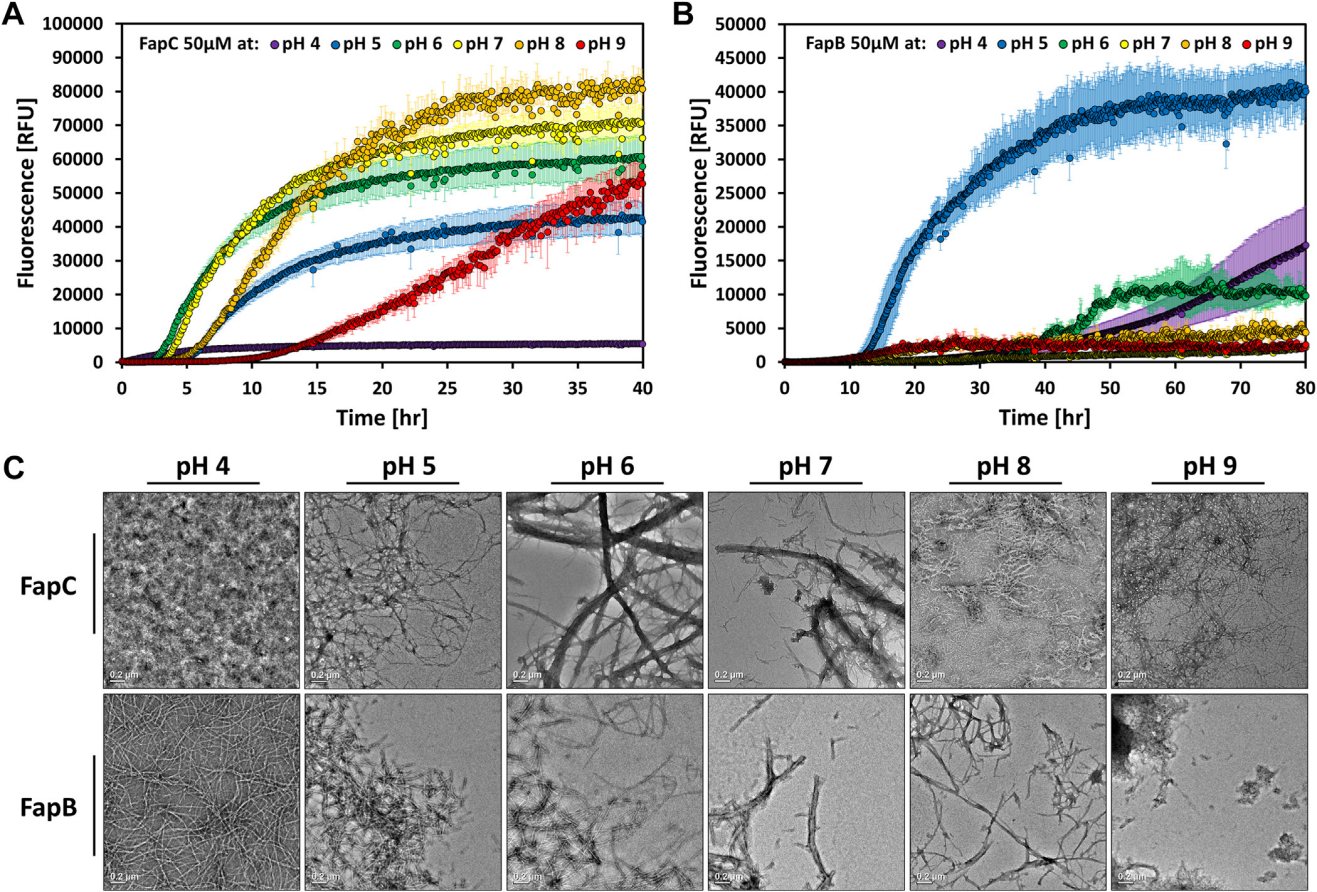
**Influence of pH on the fibrillation kinetics of histidine-tagged FapB and FapC.** ThT was used to monitor fibrillation of 50 μM histidine-tagged FapC (*A*) and FapB (*B*) between pH 4 and 9. The error bars in the graphs represent the SD from the average of triplicates. The experiments were carried out on 3 separate days with consistent results. *C*, transmission electron micrographs showcase the appearance of 50 μM FapB and FapC fibrils after 80 h of incubation under the six pH conditions, highlighting the influence of pH on fibril architecture. All micrographs include a 200 nm scale bar. Fap, functional amyloid in *Pseudomonas*; ThT, thioflavin T.

C-terminal histidine-tagged FapC, at a concentration of 50 μM, displayed a characteristic amyloid fibrillation profile, characterized by a lag phase, a growth phase, and typical sigmoidal fibrillation kinetics (Fig. 1*A*). Optimal fibrillation for FapC, with a lag phase of approximately 3 h, was observed at neutral pH values (6, 7). As pH shifted toward more acidic (pH 5) or more basic (pH 8) conditions, the lag phase increased slightly, with a marked delay at pH 9, suggesting that histidine-tagged FapC fibrillation is favored in a near-neutral environment. Notably, at pH 4, the fibrillation curve remained almost flat over a 40-h incubation period, indicating minimal fibril formation under highly acidic conditions (Fig. 1*A*). Altering the ionic strength by varying NaCl concentrations (0 M, 0.15 M, and 0.3 M) at pH 7 did not significantly impact the fibrillation curves of FapC (Fig. S3).

The fibrillation kinetics of 50 μM histidine-tagged FapB was significantly influenced by pH, exhibiting the shortest lag phases (about 10 h) at pH 5, indicating optimal fibrillation under weakly acidic conditions. At pH 4 and 6, some fibrillation was observed after a prolonged lag phase, but it was less pronounced than at pH 5. Additionally, at higher pH values (7, 8, 9), fibrillation was significantly delayed or nearly inhibited (Fig. 1*B*). Higher NaCl concentrations marginally increased fluorescence intensity but did not alter the lag phase (Fig. S3).

Of note, while analyzing the many various ThT fluorescence curves conducted in this study, including calculated slopes and maximum intensity values, our experiments revealed significant polymorphism in Fap amyloids, making it difficult to reach concrete conclusions about kinetic mechanisms. Given the high variability inherent to ThT binding across experimental conditions, extracting precise, reliable kinetic data proved challenging. For this reason, we do not discuss here detailed kinetic models such as primary or secondary nucleation or specific morphological transitions. Instead, our analysis prioritized comparing lag phases within the same experiment, for which we provide triplicate measurements and report standard error. We have ensured reproducibility by repeating experiments on different days with various protein batches, showing consistent trends in the data. While it is difficult to quantify meaningful differences in lag time statistically, we present error bars on our graphs and follow standard statistical practices for ThT assays. This approach allowed us to focus on reproducible patterns in the data, despite the inherent variability and polymorphism in the fibrillation behavior of the Fap proteins.

Transmission electron microscopy (TEM) analyses after 80 h of incubation showed the formation of fibrils for both histidine-tagged FapB and FapC under almost all pH conditions, though with differences in abundance and morphology (Fig. 1*C*). A notable distinction was observed at pH 4, where FapB formed thin, long, and well-separated fibrils, while FapC primarily produced dense bundles of aggregates where the fibrillar structure could barely be seen. In contrast, at the other extreme case of pH 9, FapC fibrils were more prevalent, indicating a preference for fibrillation in neutral to basic conditions for FapC and in acidic conditions for FapB.

Building on the established optimal pH conditions for tagged FapB (pH 5) and FapC (pH 7), we further investigated their fibrillation kinetics at additional concentrations (Fig. S4). FapC showed a dose-dependent increase in ThT fluorescence intensity at both pH levels, with a lag phase of less than 10 h at 50 μM. In contrast, FapB demonstrated rapid fibrillation with a lag phase shorter than 10 h only at pH 5, accompanied by a dose-dependent reduction in lag phase and an increased ThT fluorescence signal. However, at pH 7, FapB's fibrillation was significantly slower, with lag phase exceeding 25 h, and curves deviated from the typical sigmoidal shape, not reaching a plateau even after 70 h.

These observations underscore the distinct pH-dependent fibrillation behaviors of tagged FapB and FapC, highlighting their unique responses to environmental conditions and suggesting potential functional differences in biofilm formation and stability.

### Net charge and pH sensitivity in Fap orthologs

FapB and FapC from various *Pseudomonas* strains, including *P. aeruginosa* PAO1, *Pseudomonas fluorescens* Pf-5, *Pseudomonas putida* F1, and *Pseudomonas* sp. UK4 exhibit significant variations in length, charge, and isoelectric point, as detailed in Table S1 along with their Uniprot IDs. FapC and FapB are structured with three imperfect sequence repeats, linked together by two regions.

Additionally, structural modeling using AlphaFold indicates that these proteins adopt a Greek Key β-solenoid configuration (39, 40). The shorter linkers in FapB result in comparatively shorter sequences, likely contributing to smaller length variations among its orthologs. The sequence lengths of FapC homologs show considerable diversity, ranging from 226 amino acids in *Pseudomonas* sp. UK4 to 459 in *P. putida* F1. In contrast, the lengths of FapB homologs across these strains are more uniform, with a range from 164 amino acids in *Pseudomonas* sp. UK4 to 174 in *P. putida* F1. Despite these variations in length, FapB and FapC in the PAO1 *P. aeruginosa* strain we studied here, which are 170 and 316 residues long respectively, share a 38% sequence identity (4). This contrasts with *E. coli* CsgA and CsgB, which, despite having similar lengths (131 and 130 residues, respectively), share only 23% sequence identity.

**Figure 2:**
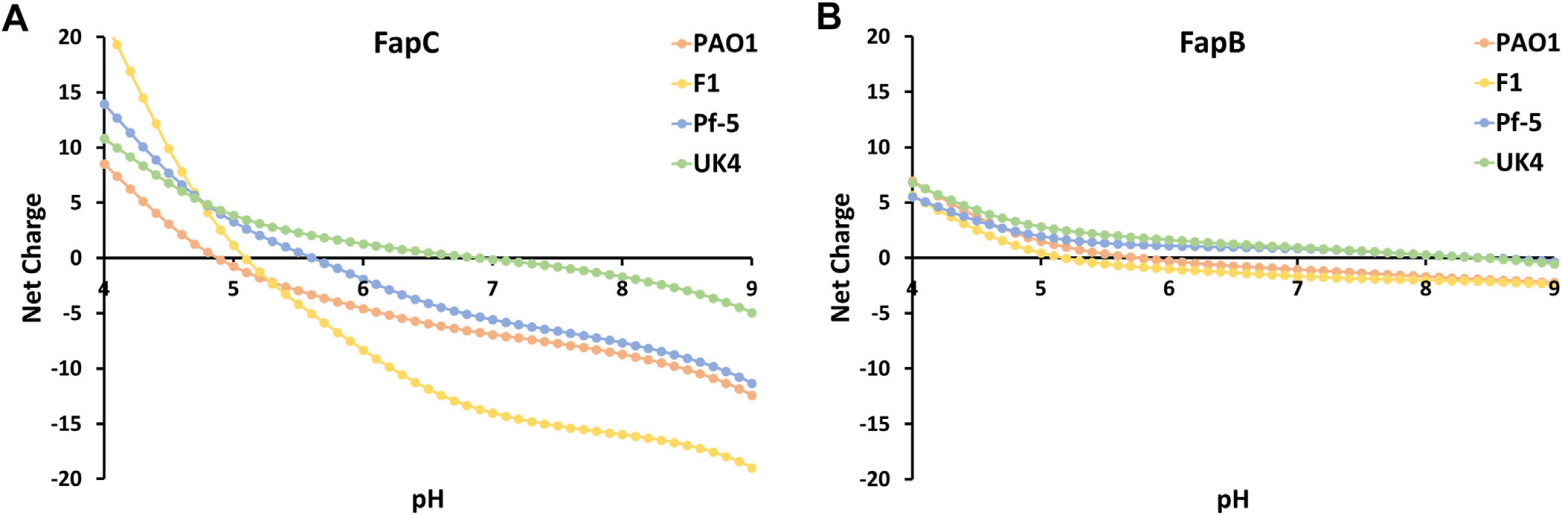
**Comparative analysis of net calculated charge for FapB and FapC orthologs.** This figure illustrates the net calculated charge of four FapC (*A*) and FapB (*B*) orthologs across a pH spectrum from 4 to 9, calculated using the Prot-pi: Protein Tool for isoelectric point and net charge calculation (https://www.protpi.ch/). Fap, functional amyloid in *Pseudomonas*.

FapB and FapC and their orthologs exhibit distinct variations in their charge distribution and sensitivity to pH. FapC orthologs, despite their different lengths, showed a narrow spectrum from slightly acidic to neutral isoelectric points, yet exhibited a broad range of total charge at neutral pH, from −14.02 to −0.13 (Table S1). The total charges of FapC orthologs were also significantly affected by pH changes (Fig. 2*A*). For example, *P. putida* F1's FapC net charge fluctuates from +20 at pH 4 to nearly −20 at pH 9, with an isoelectric point at pH 5.1 (Fig. 2*A*). FapC from *P. aeruginosa* PAO1 transitioned from a positive to a negative total charge above pH 4.9. With the addition of a histidine tag at the N- and C-terminus, this transition shifts to a higher pH of 6.1 and 5.7, respectively, with the added positive charge being mostly localized at the tag. The location of the histidine tags, whether at the N-terminus or C-terminus, did not significantly affect FapC fibrillation kinetics (Fig. S1). The fibrillation patterns were similar across pH levels 5, 7, and 9, with both tagged variants displaying rapid fluorescence increase and comparable lag phases. This suggests that the histidine tag is not likely to interfere with FapC's self-assembly, highlighting that the overall charge distribution along the protein is more crucial for fibrillation than the charge at the termini. Considering its rapid fibrillation over a wide pH range (5, 6, 7, 8), as evidenced by a lag phase of under 5 h at 50 μM (Fig. 1), this suggests that a negative charge may promote FapC fibrillation. However, at pH 9, the net charge could become overly negative, leading to repulsion that impedes orderly self-assembly. At pH 4, with a positive net charge, amorphous aggregates are formed instead of fibrils (Fig. 1*C*).

Unlike FapC orthologs, the isoelectric points of FapB orthologs exhibit slightly higher variation, spanning from acidic to basic. Despite this range, the overall charge of FapB orthologs at neutral pH remains relatively stable, oscillating between −1.64 and 0.94, as detailed in Table S1. Similarly, their net charge maintains a relatively neutral profile within a pH spectrum of 5 to 9, as shown in Figure 2*B*. Specifically, for *P. aeruginosa* PAO1 FapB, the isoelectric point is established at pH 5.8. The addition of an N- and C-terminal histidine tag shifts this point to 7.4 and 6.7, respectively, marking a more pronounced shift than that observed with FapC. Despite having a less pronounced shift in the isoelectric point, ThT fluorescence analysis revealed that FapB with a C-terminal histidine tag exhibits delayed fibrillation kinetics and significantly lower fluorescence intensity than FapB with an N-terminal tag (Fig. S2*A*). The AlphaFold-generated model of FapB monomer at varying pH levels, both with and without the tag at either end (37, 38) suggested that the surface charge distribution of this monomer remained consistent across the modeled pH levels, aligning with the predicted net charge variations (Fig. 2*B*). The C-terminal histidine tag, featuring a short linker, interacts with the surface of FapB, and likely alters the charge distribution across different pH levels, potentially explaining the linker's role in inhibiting fibrillation (Fig. S2*A*). Conversely, the model suggests that the N-terminal tag, possessing a longer linker, does not directly interact with FapB's surface, correlating with the observed fibrillation capability of FapB tagged at the N-terminus. The pH-induced charge alterations of the tag predominantly occur at the periphery of FapB due to the extended linker. FapB tagged at the N-terminus significantly promotes fibrillation at pH 5, exhibiting a lag phase of approximately 10 h at 50 μM concentration, in stark contrast to the over 30 h observed at other pH levels (Fig. 1). At this acidic pH, FapB's surface assumes a relatively neutral charge, with the linked tag carrying a positive charge.

When considering these observations in the context of the pH-dependent fibrillation lag time, it becomes clear that charge distribution plays a significant role in regulating the driving forces and rate of fibril formation and morphology for both FapB and FapC. The behavior of FapB orthologs suggests a different mechanism of aggregation compared to FapC, potentially driven by the balance of charges rather than the accumulation of a specific charge polarity.

These findings propose a sophisticated regulatory mechanism where the protein's charge significantly influences fibril formation rate and structure. Fibrillation of FapB, sensitive to pH and prone to aggregation near its isoelectric point, akin to the fibrillation observed in the peptide hormone glucagon (42), might serve as an indicator of environmental changes. In contrast, FapC, fibrillating over a broader pH range, could act as a resilient amyloid, guided by specific electrostatic interactions due to its predominantly negative charge. Such charge and pH sensitivities might represent adaptive strategies, enabling these proteins to function effectively in varied conditions within bacterial biofilms.

### Delayed fibrillation through co-incubation of histidine-tagged FapB and FapC monomers

**Figure 3:**
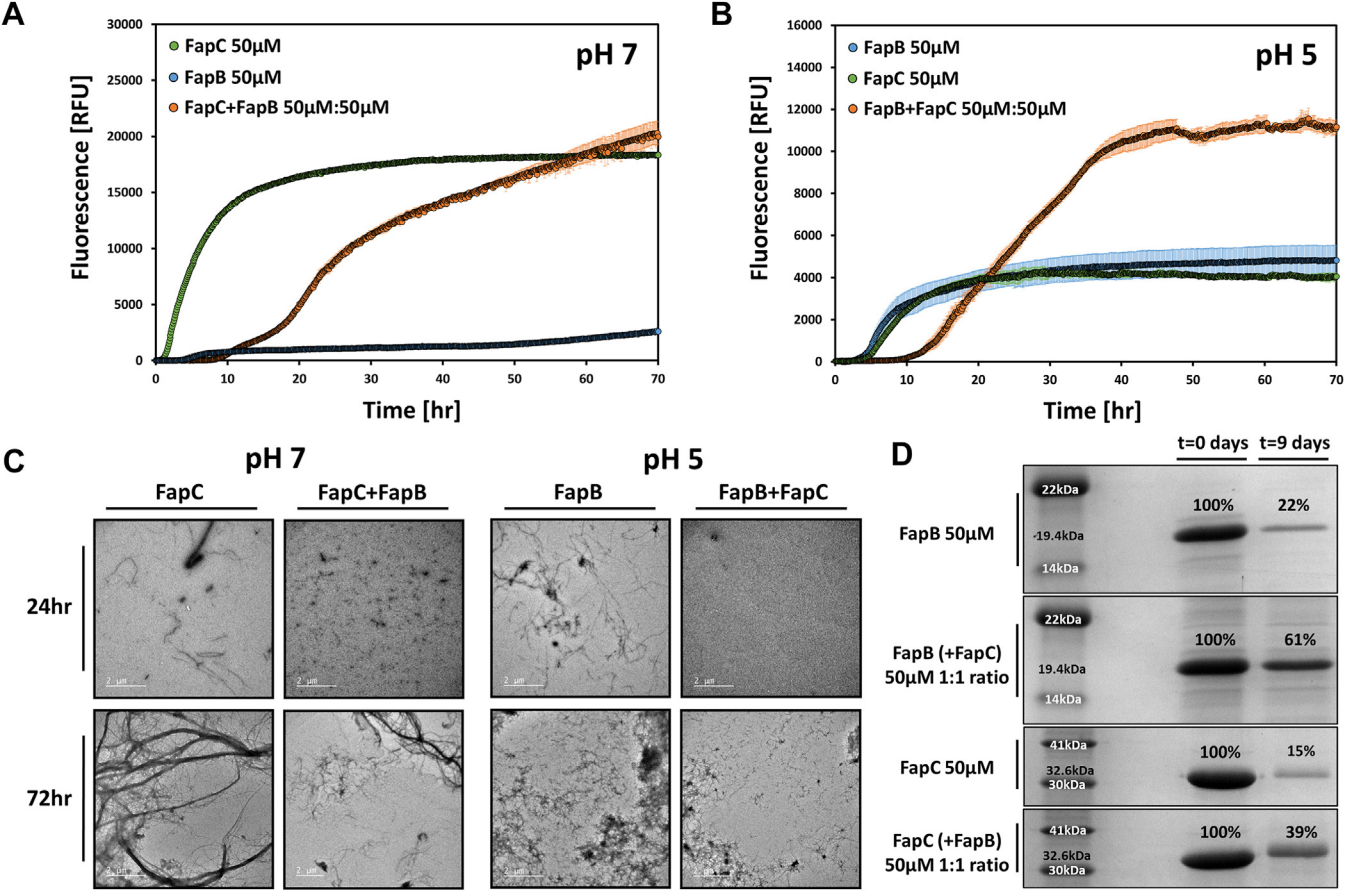
**Impact of coincubation on histidine-tagged FapB and FapC fibrillation.** ThT fluorescence shows the fibrillation kinetics of tagged FapC at pH 7 (*A*) and tagged FapB at pH 5 (*B*) both individually and when coincubated with their respective counterparts at a 1:1 M ratio (50:50 μM). Error bars indicate SD from three triplicate measurements. Repetitions of the experiment on different days yielded consistent results. The impact of varying molar ratios on fibrillation is detailed in Fig. S5. *C,* TEM images presenting 50 μM FapB (at pH 5) and FapC (at pH 7), incubated either separately or together in a 1:1 M ratio for 24 and 72 h. All images include a 2 μm scale bar for reference. *D*, SDS-PAGE analysis illustrating the remaining amounts of FapB (at pH 5) and FapC (at pH 7) monomers after 9 days of incubation, both individually and in a mixture of 1:1 M ratio. The distinct molecular weights of FapB and FapC allows for clear separation and identification of each protein on the gel. The molecular weights are indicated beside the corresponding bands for each protein. Additionally, the band intensities were quantified and presented as a percentage relative to the pre-incubation (0 day) sample, with these values displayed above each band on the gels. Fap, functional amyloid in *Pseudomonas*; TEM, transmission electron microscopy; ThT, thioflavin T.

FapB was hypothesized to act as a nucleator for the primary amyloid subunit FapC, akin to the role of CsgB with CsgA (8, 11, 20, 21, 43). To investigate their potential reciprocal effects on fibrillation, we introduced various concentrations of fresh (0 h incubation)-tagged Fap monomers (from 2 μM to 100 μM) into a solution containing 50 μM of their counterparts, maintaining the optimal pH for each (Fig. S5, *A* and *B*). Fig. S5, *C* and *D* compare the fibrillation kinetics of tagged Fap monomers incubated alone and as mixtures in both pHs and highlights three specific molar ratios (1:1.5, 1:1, and 2:1), while Figure 3, *A* and *B* showcases the results for a representative 1:1 M ratio. The coincubation of Fap monomers resulted in a dose-dependent inhibitory effect, notably prolonging the lag phase of their fibrillation.

The addition of FapB to FapC at pH 7 resulted in a dose-dependent extension of the lag phase compared to FapC fibrillation alone (Figs. 3 and S5). This delay increased from a lag phase of approximately 2 h to as much as 15 h with increasing concentrations of FapB. At 75 μM, FapB shows rapid fibrillation even at pH 7, with a lag phase of approximately 15 h, which seems to nevertheless halt the fibrillation lag time of the 50 μM FapC (Fig. S5*C*). This suggests that FapB, which fibrillates more slowly, increases the fibrillation lag time of FapC at a range of concentrations. At pH 5, a comparable dose-dependent inhibitory effect was observed, with the lag phase of FapB extending from about 5 h to as long as 25 h following the addition of FapC (Fig. S5, *B* and *D*). Despite the significant fibrillation of FapC alone also at pH 5 (Fig. S5*D*), its addition reduced the fibrillation lag time of FapB (Fig. 3*B*).

The bidirectional delay in fibrillation was visually confirmed by TEM images, which showed a reduced number of fibrils in the mixed samples after 24 h of incubation at 50 μM 1:1 M ratio at both pH levels. However, after 72 h, this inhibition appeared to diminish, and fibrils formed in both mixtures, consistent with the kinetic assay results (Fig. 3*C*). To quantitatively assess the extent of fibrillation, we exploited the resistance of Fap fibrils to SDS and boiling (8, 11, 44, 45) and measured the levels of residual monomers using SDS-PAGE before and after 9 days of incubation, both individually and in 1:1 mixtures (Fig. 3*D*). The notable difference in molecular weight between the Fap proteins enabled precise detection of each individual Fap level, even when mixed and run together in the gel. Analysis showed a higher monomer concentration in the mixed amyloid samples than in those where Faps were incubated separately, at both pH levels. Specifically, for FapC, after 9 days, the monomer concentration was 15% of the preincubated sample when incubated alone, and 39% when mixed in a 1:1 ratio with FapB. In the case of FapB, the monomer concentration after 9 days was 22% of the preincubated sample when incubated alone, and 61% when mixed in a 1:1 ratio with FapC. Overall, the data indicate that mixing different Fap proteins tends to decelerate their fibrillation process, with lasting effects even after 9 days. The extent of this effect varies based on the specific Fap combination used, with FapC demonstrating a more pronounced influence on FapB.

### Self-seeding and cross-seeding experiments of histidine-tagged FapB and FapC reveal asymmetrical effects on fibrillation

**Figure 4:**
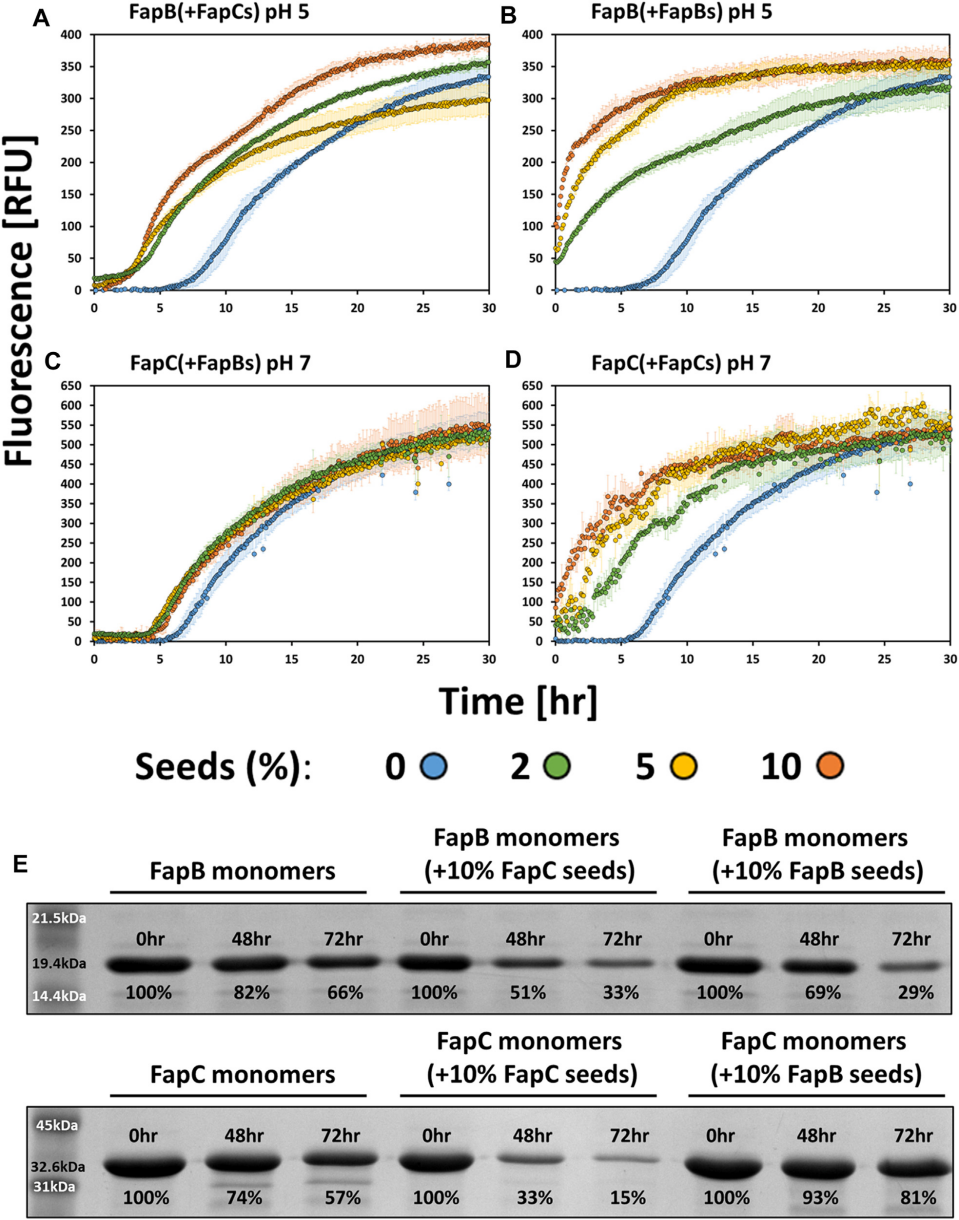
**Effect of self-seeding and cross-seeding on the fibrillation of histidine-tagged Faps.***A–D*, ThT fluorescence assays demonstrate the impact of adding fibril seeds (indicated as “s” in the figure) of either tagged FapB or FapC on the fibrillation kinetics of 50 μM tagged FapB at pH 5 (*A* and *B*) and 50 μM tagged FapC at pH 7 (*C* and *D*). Seeds were introduced at three different volume percentages of the total reaction mixture (2%, 5%, and 10%), and the fibrillation process was monitored over 30 h. The experiments were conducted with FapB in the presence of FapC seeds at pH 5 (*A*), FapB in the presence of FapB seeds at pH 5 (*B*), FapC in the presence of FapB seeds at pH 7 (*C*), and FapC in the presence of FapC seeds at pH 7 (*D*). Seed concentrations are color-coded: 0% (*blue*), 2% (*green*), 5% (*yellow*), and 10% (*orange*). Error bars represent the SD from triplicate measurements. Replications on different days consistently produced similar trends. *E*, SDS-PAGE analysis of FapB and FapC monomers following incubation with or without fibril seeds. The *top panels* display the remaining amounts of 50 μM FapB monomers at 0, 48, and 72 h when incubated alone or with 10% v/v FapC or FapB seeds. The *bottom panels* show the corresponding analysis for 50 μM FapC monomers under similar conditions (incubated alone or with 10% v/v FapC or FapB seeds). Molecular weight markers are indicated. The distinct molecular weights of FapC (∼32.6 kDa) and FapB (∼19.4 kDa) allow for their clear separation and identification in the gels. Quantification of the band intensities, shown as a percentage of the initial monomer amounts (0 h), is displayed beneath each band, highlighting the impact of seeding on fibril formation over time. Fap, functional amyloid in *Pseudomonas*; ThT, thioflavin T.

To better understand the interactions between these two proteins, we performed self-seeding and cross-seeding tests using preformed, sonicated fibrils of each protein. The effect of the seeds on the fibrillation rates of 50 μM tagged FapB or FapC monomers was evaluated by adding 2 to 10% of the seeds (Fig. 4). Self-seeding accelerated fibrillation for both FapB and FapC, with FapB showing a pronounced reduction in its fibrillation lag phase, nearly eliminating it (Fig. 4*B*). SDS-PAGE analysis with 10% seeds (Fig. 4*E*) confirmed that self-seeded FapB and FapC monomers were cleared more rapidly than unseeded samples after 48 or 72 h of incubation, reinforcing the enhanced fibrillation even after prolonged incubation.

Cross-seeding experiments revealed that FapC seeds reduced the lag phase of FapB fibrillation in a dose-dependent manner (Fig. 4*A*). SDS-PAGE analysis supported these findings, with FapB monomers cross-seeded with 10% FapC seeds showing reduced residual monomers indicating enhanced fibrillation even after few days of incubation. However, FapC showed little to no change in its lag phase when seeded with FapB seeds at any tested concentration. The SDS-PAGE analysis showed that FapB seeds led to slight increased residual monomers of FapC, suggesting a minimal or even inhibitory effect on FapC fibrillation over 48 to 72 h.

Overall, the results suggest that while self-seeding promotes fibrillation in both FapB and FapC, cross-seeding effects are asymmetrical. FapC seeds notably enhance the fibrillation of FapB, as seen by shorter lag phases and reduced monomer content over time. However, FapB seeds have only a slight effect on FapC fibrillation, shortening the lag phase marginally and not significantly affecting long-term fibril formation. These results highlight an asymmetry in the interactions between FapB and FapC, suggesting that FapB may not serve as a strong nucleator for FapC in this context, particularly in this tested strain of *P. aeruginosa PAO1*. Instead, FapB and FapC may follow different nucleation and elongation pathways during amyloid formation, contributing to their distinct roles in biofilm development and environmental adaptation. This suggests more complex interactions between these amyloids than previously understood, warranting further exploration into their specific functions within *Pseudomonas* biofilms.

### Fluorescence microscopy reveals colocalization of histidine-tagged FapB and FapC

**Figure 5:**
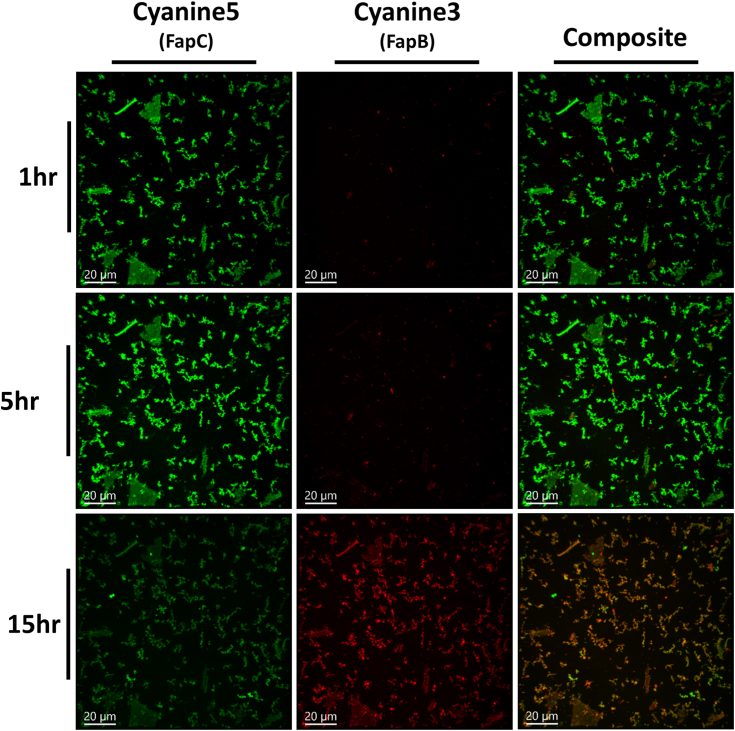
**Dynamic visualization of histidine-tagged FapB and FapC monomers colocalizing over time.** Fluorescence microscopy images, captured from Video S1, with FapC labeled with Cyanine5 (visualized in *green*) and FapB labeled with Cyanine3 (visualized in *red*). FapB and FapC are coincubated as fresh samples. Composite images show the merging of Cyanine3 and Cyanine5 channels result in a *yellow/orange hue*, pinpointing the areas where FapB and FapC aggregates intersect. The images capture the progression of aggregation and colocalization at 1, 5, and 15 h, arranged in a *top-to-bottom sequence*. Incubation during the time-lapse imaging was conducted at pH 7. Consistently included in all images are scale bars, each denoting a length of 20 μm. Fap, functional amyloid in *Pseudomonas*.

**Figure 6:**
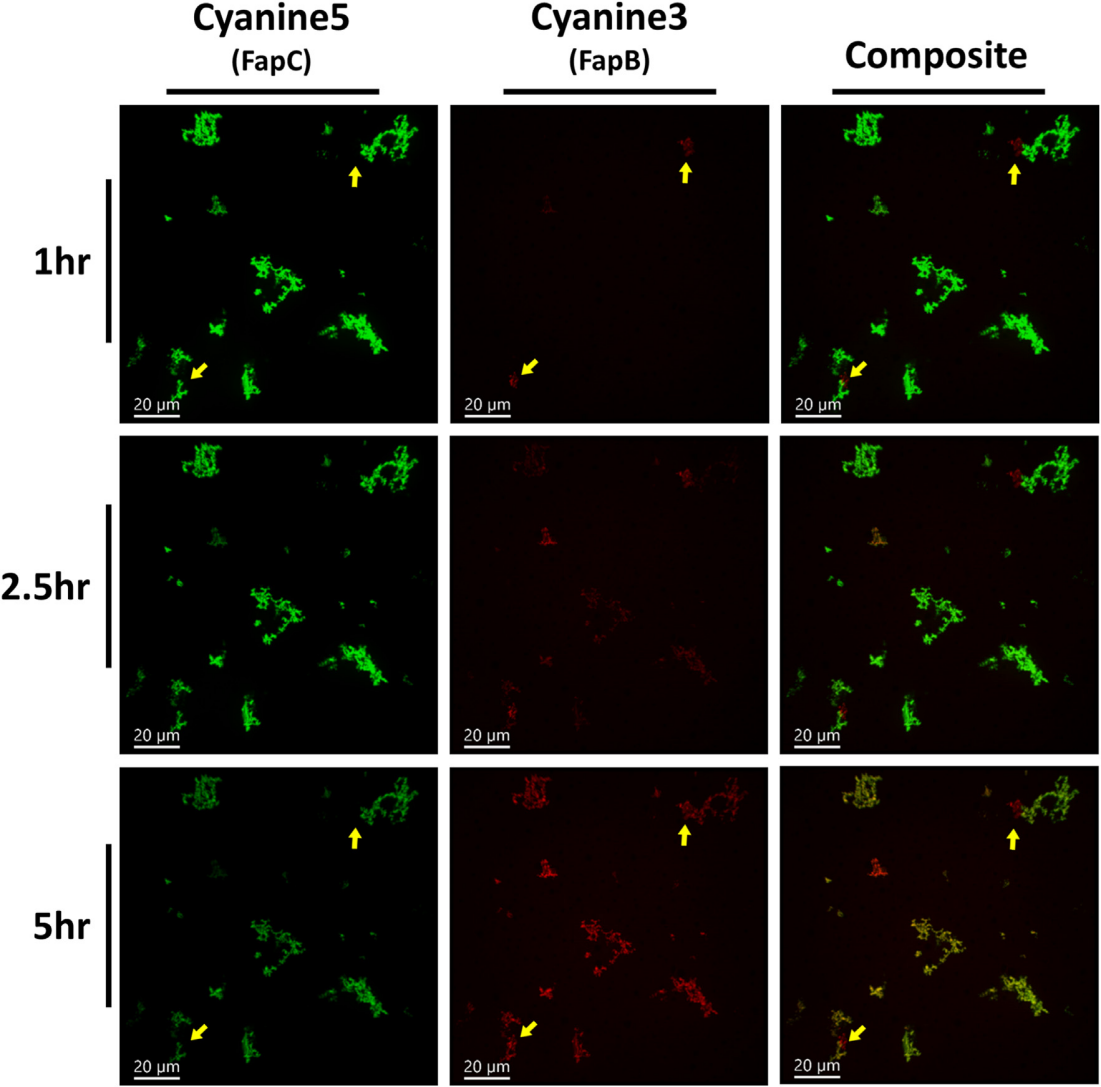
**Dynamic visualization of histidine-tagged FapB and FapC preincubated samples colocalizing over time.** Fluorescence microscopy images, captured from Video S2, with 24 h separately preincubated FapC labeled with Cyanine5 (visualized in *green*) and FapB labeled with Cyanine3 (visualized in *red*), and coincubated for 6 h. The images capture the progression of colocalization at 1, 2.5, and 5 h. Composite images show the merging of Cyanine3 and Cyanine5 channels, producing a *yellow/orange hue* that pinpoints areas where FapB and FapC aggregates intersect. *Yellow arrows* are used to pinpoint aggregates of preformed FapB, highlighting their minimal colocalization with FapC over the observed time frame. Incubation during the time-lapse imaging was conducted at pH 7. Scale bars, consistently included in all images, represent a length of 20 μm. Fap, functional amyloid in *Pseudomonas*.

Alterations in fibrillation kinetics observed during the coincubation of Fap monomers point to a direct interaction between them. To further explore this, we employed confocal light microscopy for in-depth visualization. FapB and FapC were labeled with Cyanine3 and Cyanine5 fluorophores, respectively, using N-hydroxysuccinimide (NHS) ester crosslinking to attach these dyes to lysine residues within the proteins. The fibrillation of these labeled proteins was verified through TEM (Fig. S6). Our analysis utilized two distinct approaches: first, we observed the aggregation and localization of fresh (∼0–1 h incubation) monomeric Faps (Fig. 5 and Video S1); second, we examined the interactions between preformed (∼24 h preincubation) fibrils (Fig. 6 and Video S2). Due to resolution constraints, the observed foci were referred to as aggregates, without distinguishing between fibrils and less ordered species.

Over a 20-h time-lapse imaging period, we monitored the formation and localization of aggregates from a 50 μM mixed solution of FapB and FapC at pH 7. Within the first hour, significant FapC aggregates formed, some reaching up to 15 μm, while FapB aggregates were smaller. This observation is consistent with kinetic and TEM assays, which indicated a slower fibrillation kinetics for FapB than FapC, especially at pH 7. After 10 h, there was a notable increase in FapB aggregate formation, primarily colocalized with FapC, intensifying by the 15th h (Fig. 5 and Video S1). The current resolution limits our ability to differentiate between the formation of mixed hetero-fibrils and entangled individual protofilaments.

In our second experimental setup, we explored the dynamics of preformed Fap fibrils, which had been incubated individually for 24 h, by mixing them together for a 6-h time-lapse imaging period (Fig. 6 and Video S2). Initially, our observations revealed a predominance of FapC aggregates and a lesser quantity of FapB aggregates (indicated by yellow arrows in Fig. 6), with minimal colocalization between them. As the experiment progressed, we noted a notable increase in the colocalization of FapB aggregates with the preexisting FapC aggregates. Interestingly, the FapB aggregates that had formed prior to the mixing did not show colocalization with the newly formed FapC aggregates. This observation underscores an asymmetrical interaction pattern between FapB and FapC, where FapC appears to have a more pronounced influence on the behavior of FapB, rather than the other way around. This asymmetry in Fap interaction suggests a directional influence in their aggregation behavior, with FapC playing a more dominant role. To mitigate the potential impact of photobleaching of the Cyanine5 dye on FapC amyloids, control samples were prepared under identical conditions but only visualized at the endpoint. These controls exhibited a similar colocalization pattern in both monomer and preformed fibril experiments (Fig. S7).

**Figure 7:**
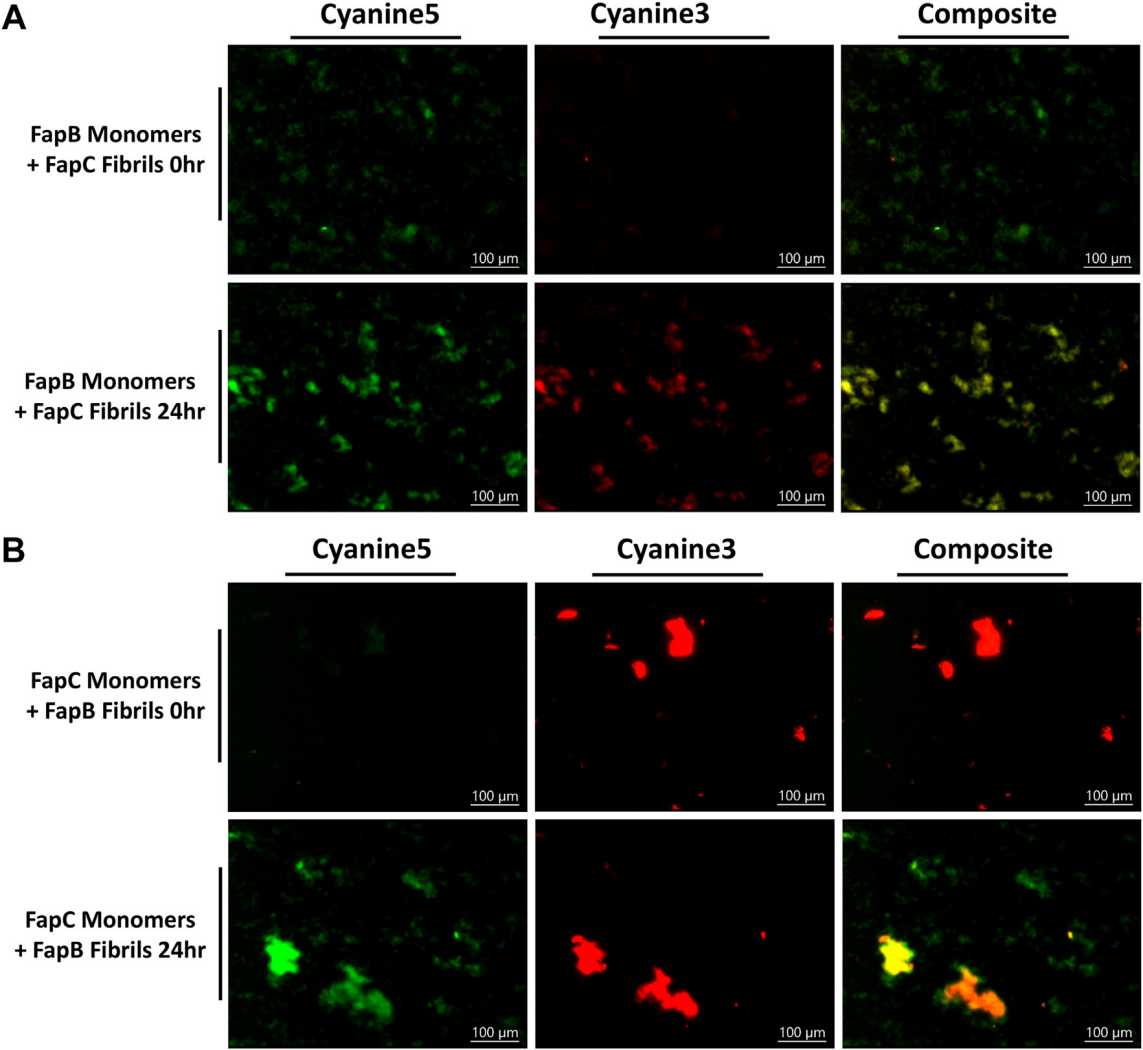
**Visualization of colocalization of histidine-tagged FapB and FapC monomers with fibrils over time.** Fluorescence microscopy images show FapC labeled with Cyanine5 (*green*) and FapB labeled with Cyanine3 (*red*), coincubated with one protein in fibrillar form and the other in monomeric form. *A*, displays the colocalization of FapB monomers with FapC fibrils, while (*B*) shows the colocalization of FapC monomers with FapB fibrils. Images were captured immediately after mixing (0 h) and after 24 h of incubation. The *left column* represents the Cyanine5 channel (FapC), the *middle column* shows the Cyanine3 channel (FapB), and the *right column* combines both channels. *Yellow/orange regions* in the composite images highlight areas where monomers intersect with fibrils. Scale bars represent 100 μm. Fap, functional amyloid in *Pseudomonas*.

Further investigation into the colocalization of FapB and FapC monomers with fibrils over time provided additional evidence of their asymmetric interaction (Fig. 7). The data show that FapB monomers rapidly associate with preformed FapC fibrils, as indicated by increased colocalization after 24 h (Fig. 7*A*). In contrast, FapC monomers exhibit only limited colocalization with preformed FapB fibrils, even after 24 h of incubation (Fig. 7*B*). These findings highlight a preferential interaction where FapB monomers are more likely to bind with FapC fibrils, while FapC monomers show limited incorporation into FapB fibrils.

This suggests that FapC fibrils may serve as nucleation sites or templates for FapB, whereas FapB fibrils are less effective at incorporating FapC monomers. These observations are consistent with the asymmetric effects noted in the self-seeding and cross-seeding experiments (Figs. 3 and 4). Both experiments indicate that FapC has a stronger influence on the aggregation behavior of FapB, whether through direct fibril–monomer interactions or seeding effects. The limited impact of FapB on FapC, observed in both colocalization and seeding studies, reinforces the idea that FapB is more readily incorporated into structures nucleated by FapC.

This preferential interaction may be attributed to structural differences between FapB and FapC fibrils or specific surface properties that promote selective binding. Understanding these selective interactions is crucial, as they could affect the structural integrity and functional properties of amyloids in biofilms, potentially influencing biofilm resilience and resistance to environmental stressors.

### Structural insights into FapB and FapC Fibril formation using AlphaFold3

**Figure 8:**
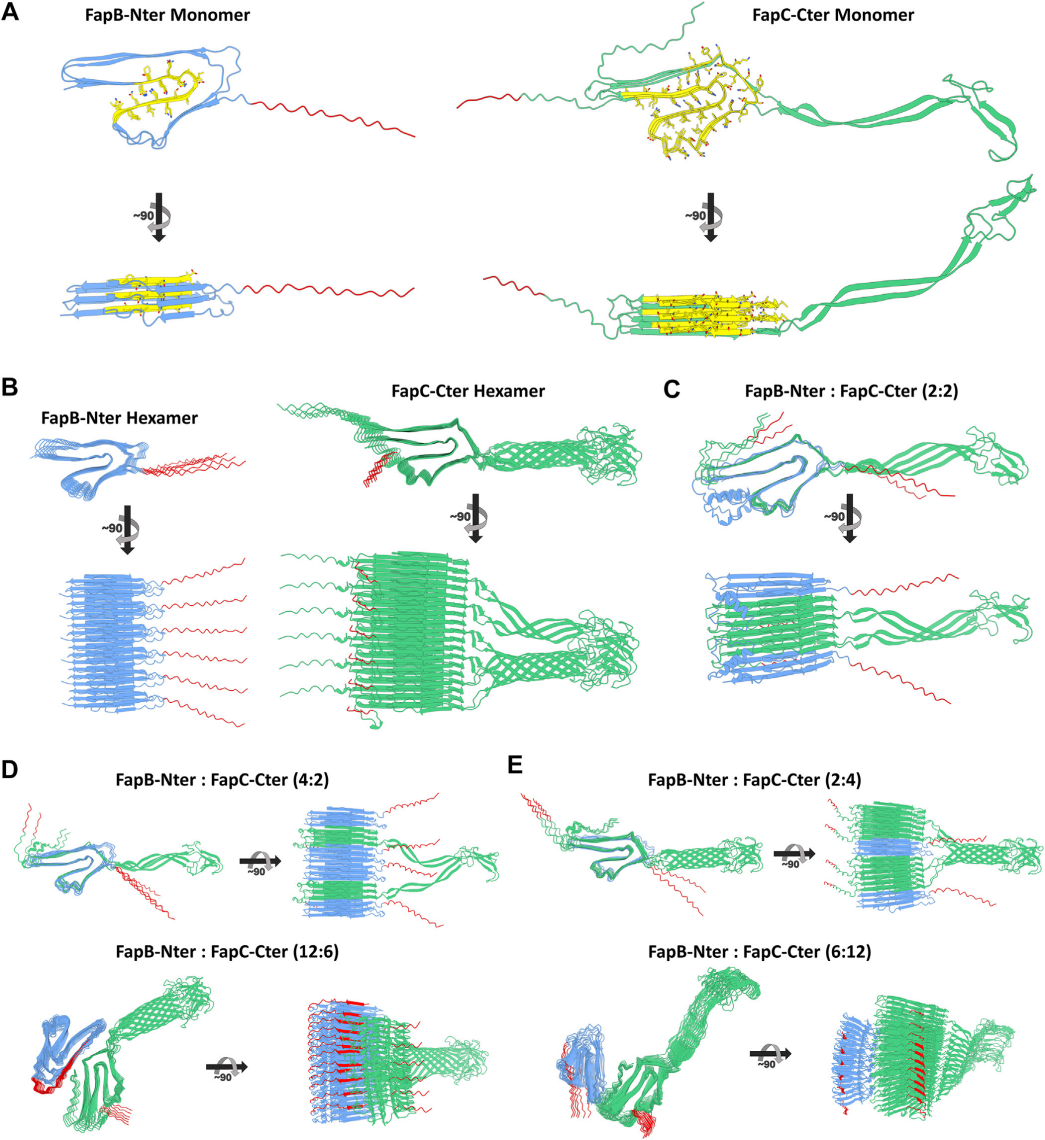
**AlphaFold3 predicted structures of FapB and FapC amyloid monomers and multimers with histidine tags.** Top-ranked AlphaFold3 models of *Pseudomonas aeruginosa* PAO1 FapB (*blue*) and FapC (*green*), including the histidine tags used in this study at either the N-terminal (NterHis) or C-terminal (CterHis) sites, shown in *red*. Each panel presents the amyloid structures from both a *top view* (*top row*) and a *side view* after a ∼90° rotation (*bottom row*). *A*, predicted monomeric structures of FapB-NterHis and FapC-CterHis. Regions with a Greek key β-solenoid amyloid core are shown in *yellow*, with side chains displayed as *sticks*. *B*, hexameric structures of FapB-NterHis (*left*) and FapC-CterHis (*right*). *C*, FapB-NterHis and FapC-CterHis in a 2:2 stoichiometric ratio, predicting the formation of a mixed fibril. *D* and *E*, the Faps in 4:2 (*top*) and 12:6 (*bottom*) stoichiometric ratios, showing how varying proportions affect the fibril structure. Fap, functional amyloid in *Pseudomonas*.

Until high-resolution structures of Faps and their complexes are available, we used AlphaFold3, a cutting-edge artificial intelligence (AI)-based structural modeling tool, to predict the monomeric and multimeric structures of *P. aeruginosa* PAO1 FapB and FapC, both with histidine tags. The predictions revealed characteristic Greek key β-solenoid structures, featuring β-strands with three imperfect sequence repeats connected by two linker regions, aligned perpendicular to the fibril elongation axis (Fig. 8). These results align with previous studies, emphasizing the role of imperfect repeats in FapB and FapC in promoting amyloid formation and structural stability (4, 8, 23, 39, 40). Notably, FapC's linker regions remain partially unstructured and outside of the fibril core. To explore the interactions between FapB and FapC, we modeled various ratios of subunits (Fig. 8, *C–E*). At lower subunit numbers, the proteins tended to form linear, copolymer-like fibrils, allowing continuous elongation. However, with an increasing number of units, fibrils formed separately and bundled together, reflecting a shift in interaction dynamics. This increase in complexity reduced model accuracy, as seen by more frequent collisions, highlighting the limitations of the algorithm in predicting highly multimeric structures (Fig. 8, *D* and *E*, bottom panels). The inclusion of His-tags in these models had minimal impact on fibril assembly and function, indicating that their presence does not significantly alter the overall structure. Together with our kinetic and microscopic observations, these predictions suggest that FapB and FapC form intermolecular interactions leading to distinct fibrillar states. Further structural studies are needed to better understand the formation of linear fibrils composed of both proteins, as well as the bundling of separate fibrils.

### Histidine-tagged FapB and FapC fibrils exhibit thermostability under extreme heat conditions

**Figure 9:**
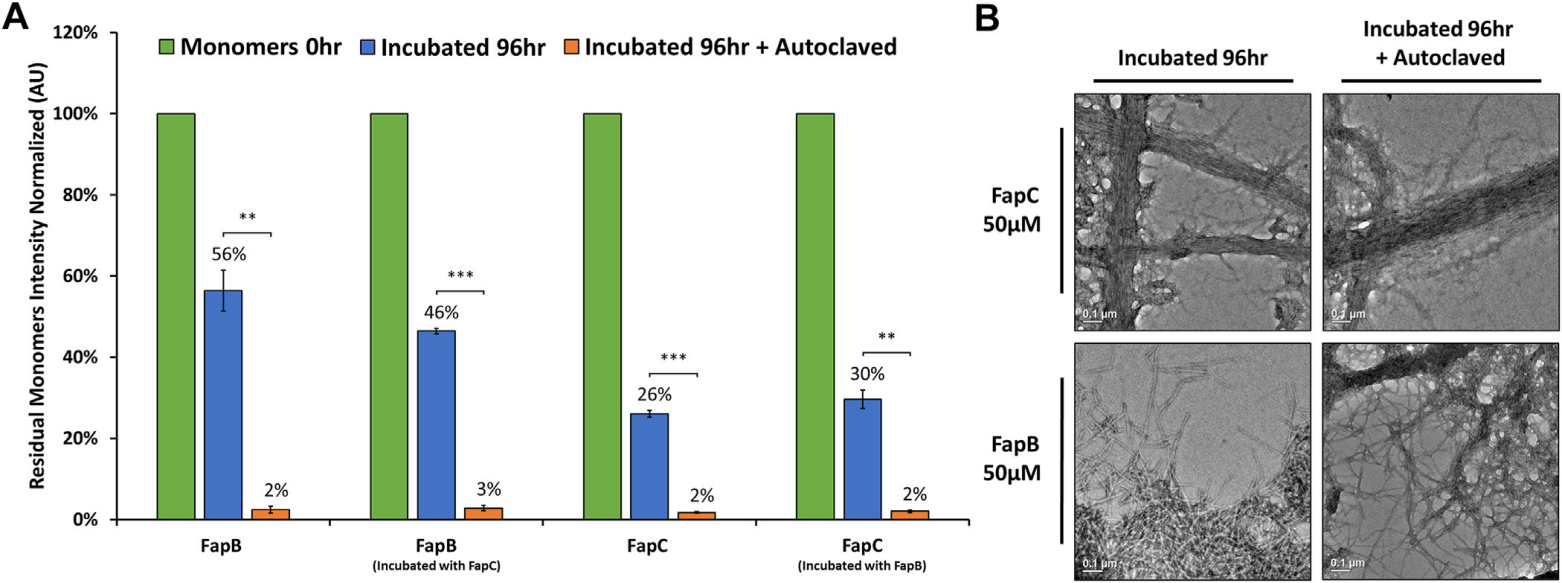
**Resilience of histidine-tagged FapB and FapC fibril formation under extreme high-temperature autoclaving conditions.***A*, quantification of SDS-PAGE band intensities of 50 μM FapB (incubated at pH 5) and 50 μM FapC (incubated at pH 7) monomers, after incubation with or without the counterpart protein at a 1:1 ratio. The columns represent the remaining monomers after 96 h of incubation, with and without autoclave treatment, shown as a percentage of their initial amount at 0 h. The results represent the average of three independent experimental repetitions (one of the gels is shown in Fig. S10). Statistical significance was assessed using a one-way ANOVA, followed by Tukey’s *post hoc* test (details in the Methods section), comparing the 96-h autoclaved samples against the 96-h nonautoclaved samples. *Asterisks* indicate levels of statistical significance: ∗∗ for *p* < 0.01 and ∗∗∗ for *p* < 0.001. *B*, TEM micrographs display FapB and FapC after 96 h of incubation, both before and after autoclave treatment. Scale bars in all micrographs represent 0.1 μm. Fap, functional amyloid in *Pseudomonas*; TEM, transmission electron microscopy.

Due to their potential scaffolding role in biofilms, we evaluated their thermal stability. FapB and FapC were incubated at 37 °C for 4 days at pH 5 and 7, respectively, to form fibrils. These fibrils were then exposed to a heat treatment at 85 °C for 6 and 24 h (Fig. 9). The residual monomers were quantified using SDS-PAGE, taking advantage of the fibril resistance to both SDS buffer and boiling (8, 11, 15, 19, 45) (Fig. S8). Additionally, due to the difference in molecular weights—FapB at 19.4 kDa and FapC at 32.6 kDa (Table S1)—we analyzed each protein separately to evaluate their respective band intensities.

As expected by Fap fibrillation kinetics, the 4-day incubation period resulted in a significant reduction in the quantity of monomers (Fig. 9*A*). Additionally, the data indicated that incubated FapC had fewer residual monomers than FapB (26% and 56%, respectively), suggesting a more robust fibrillation of FapC. The presence of FapB did not significantly change the level of FapC monomers (30%). The presence of FapC however, further decreased the level of FapB monomers during 4-day incubation to 46%, suggesting inhibitory effect on fibrillation, as observed by the kinetics measurements and confocal microscopy visualization. Post heat treatment for both 6 and 24 h eliminated detectable monomers of both FapB and FapC samples (Fig. S8), indicating further fibrillation. Confocal microscopy images corroborated the findings, showing increased aggregation of FapB and FapC over time, while heat treatment at 85 °C further promoted the formation of larger aggregates (Fig. S9).

The experiment was extended to more extreme conditions by autoclaving the preformed fibrils for 20 min at 121 °C. SDS-PAGE analysis revealed a dramatic reduction in monomeric content, with FapB and FapC showing only 2 to 3% residual monomers, further confirming their high propensity for fibrillation under extreme heat. One exemplary SDS-PAGE is presented in Fig. S10, and quantification of band intensity with statistical values based on three different experiments is presented in Figure 9*A*. TEM micrographs (Fig. 9*B*) showed that these fibrils remained largely intact even after autoclaving, demonstrating their remarkable thermostability.

Overall, the findings underscore the extraordinary thermostability of Fap fibrils, suggesting that heat shokes, and even autoclaving, while likely effectively kill bacterial cells, may not be sufficient to completely eliminate the Fap fibrils themselves.

### Chemical stability of histidine-tagged FapB and FapC fibrils

**Figure 10:**
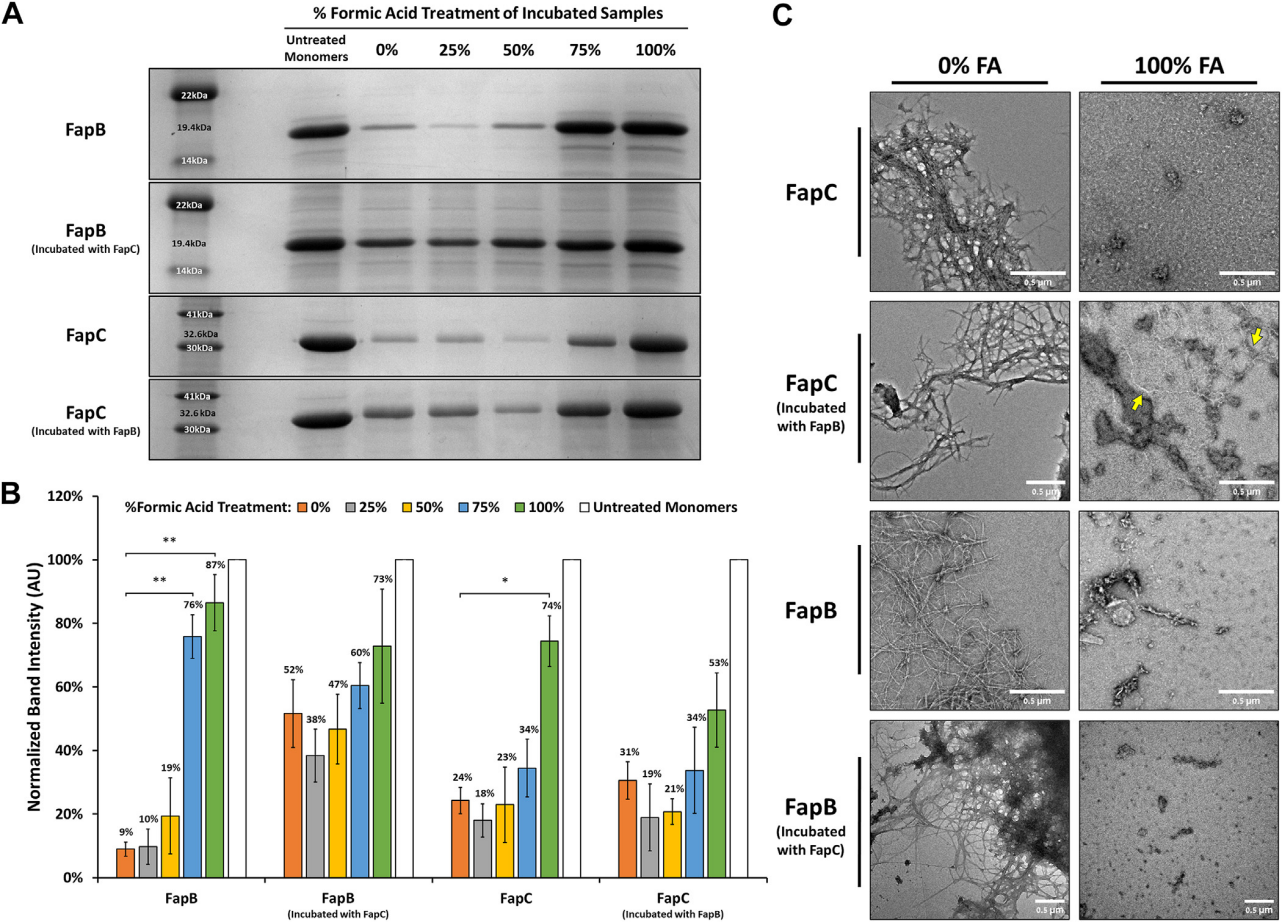
**The stability of histidine-tagged FapB and FapC fibrils in the presence of formic acid.***A*, representative SDS-PAGE gels showing FapB (at pH 5) and FapC (at pH 7) after a 9-day incubation period, either separately or together in a 1:1 ratio, followed by exposure to increasing concentrations of 25%, 50%, 75%, and 100% formic acid (FA). Molecular weight markers are included. *B*, quantification of band intensities from SDS-PAGE analysis averaged across three independent experiments, including the one shown in *panel A*. The graph presents the normalized fraction of soluble monomers relative to their respective untreated monomers (0 h) controls. Error bars indicate the SD from the mean of three experiments conducted on different days. Statistical significance was assessed using a one-way ANOVA, followed by Tukey’s *post hoc* test (details in the Methods section), comparing each treatment against the untreated monomers (0 h) controls. *Asterisks* indicate levels of statistical significance: ∗ for *p* < 0.05 and ∗∗ for *p* < 0.01. *C*, TEM micrographs depicting the structural integrity of FapB and FapC fibrils after a 9-day incubation, separately or together in a 1:1 ratio, followed by treatment with 0% or 100% FA. The images reveal the visual changes in fibril structure and integrity, with 0% FA-treated samples showing intact fibril networks, while 100% FA-treated samples exhibit disrupted and fragmented fibrils. *Yellow arrows* highlight specific areas of fibril fragmentation that showed resistance in the 100% FA-treated samples. Scale bars represent 0.4 μm. Fap, functional amyloid in *Pseudomonas*; TEM, transmission electron microscopy.

The chemical stability of FapB and FapC fibrils was investigated, given the known resilience of amyloid fibrils to various chemicals like SDS, urea, and guanidinium chloride (19, 45). Notably, both CsgA and FapC fibrils can be dissolved into monomers by high concentrations of FA (4, 19, 45, 46, 47). We used increasing concentrations of FA to assess the stability of FapB and FapC fibrils, incubated individually and together for 9 days, at pH 5 and 7, respectively. The analysis involved quantified SDS-PAGE band intensities to detect any residual monomers after FA treatment normalized against untreated samples (Fig. 10).

FapB fibrils demonstrated a notable resistance to FA up to a concentration of 50%, with no significant change in the percentage of monomers. However, at 75% FA, a marked disaggregation was observed, resulting in 76% of residual monomers compared to the control pre-incubated samples, a significant increase from the 9% observed without FA. This disaggregation further escalated at undiluted FA, with 87% of the control monomers present. TEM images of FapB confirmed abundant fibril formation after incubation and then significant disaggregation at undiluted FA (Fig. 10*C*). When FapB was coincubated with FapC, they exhibited greater solubility than FapB incubated alone, with 52% residual monomers. The addition of FA did not significantly affect the percentage of remaining monomers, with residual monomers ranging up to 73% (Fig. 10*B*). These findings corroborate the inhibitory effect of FapC on the fibrillation of FapB, as observed in the kinetic assays (Fig. 3). Furthermore, the TEM images of FapB coincubated with FapC at pH 5 show that despite the high amount of monomers present compared to FapB incubated alone, fibrils do form, which then disaggregate at 100% FA (Fig. 10*C*).

FapC displayed remarkable chemical stability, maintaining 34% residual monomers even after the addition of 75% FA, compared to 24% without FA (Fig. 10*B*). With undiluted FA, the soluble fraction was only 74%, indicating the presence of some still resistant fibrils. Interestingly, the presence of FapB did not significantly affect FapC's stability, with similar percentages of monomers with and without FapB up to 75% FA. Yet, at undiluted FA, the soluble fraction of FapC with FapB was only 53%. TEM micrographs showed thin fibrils of FapC with FapB at pH 7, even with undiluted FA (Fig. 10*C*), supporting a stabilizing effect of FapB on FapC fibrils at this extreme condition.

Comparatively, the mutual effects of FapB and FapC were contrasted with those of curli amyloids CsgA and CsgB (Fig. S11). Coincubation of CsgA and CsgB did not inhibit fibrillation, aligning with CsgB's role as a nucleator for CsgA. However, their mixed fibrils were less chemically stable than those formed separately, as indicated by higher monomer counts at lower FA percentages. These observations highlight fundamental differences between the curli and Fap systems in terms of the interactions and mutual effects between their amyloid components.

## Discussion

### Differential charge and pH-dependence of histidine-tagged Fap amyloids: Implications for environmental adaptation

The distinct charge characteristics and pH sensitivities of FapB and FapC fibrillation highlight their potential roles in environmental adaptability. Drawing parallels with amyloid-β in Alzheimer's disease, previous studies have shown how environmental factors like pH, ionic strength, temperature, and polypeptide concentration, along with interamyloid interactions, critically influence amyloid functions, pathologies, and structures (13, 48, 49, 50, 51, 52). The unique pH sensitivities of FapB and FapC underscore their varied interactions with environmental conditions, marked by differences in net charges, isoelectric points, and conservation across orthologs (Table S1 and Fig. 2). Their fibrillation responses to pH shifts in PAO1 tested here further emphasize these distinctions (Figs. 1 and S4).

This diversity in physical and chemical properties among the homologs of FapB and FapC across different *Pseudomonas* strains highlights the complexity and variability of these biofilm-forming proteins. The fibrillation of FapB, which is sensitive to pH and driven by aggregation around its isoelectric point, may even serve as an indicator of environmental changes, and perhaps a preference for different living conditions among various strains. For instance, FapB from *P. aeruginosa* PAO1 and *P. putida* F1 shows a transition from positive to negative net charge around pH 5, whereas FapB from *Pseudomonas* sp. UK4 and *P. fluorescens* Pf-5 only undergoes this switch at pH levels above 9 (Fig. 2). This characteristic could be the key in regulating fibrillation in vastly different environmental conditions. On the other hand, FapC appears to function as a more robust amyloid, capable of fibrillating across a wide pH range. Its fibrillation process is driven by specific electrostatic interactions that are dependent on its net negative charge. This suggests that the ability of FapC to form fibrils is less influenced by pH changes compared to FapB. Such charge and pH sensitivities, along with higher stability, might represent adaptive strategies, enabling these proteins to function effectively in varied conditions within bacterial biofilms.

*Pseudomonas* species, especially *aeruginosa*, thrive in diverse environments like soil, water, plants, animals, and sewage, which vary significantly in pH, temperature, and salinity (4). For instance, environmental pH can range from 4.5 to 9.5, demanding substantial bacterial adaptability (4, 53). The optimal conditions for FapC PAO1 fibrillation (pH 6–7) align with the environments in lungs and airways, particularly in CF patients (4, 54, 55). Moreover, the more alkaline environment in chronic wounds may favor FapC fibrillation, contributing to persistent infections (4, 54, 55). Conversely, the preferred conditions for FapB PAO1 fibrillation (pH 5) correlate with the acidity in severely inflamed tissues (56), suggesting a distinct role for FapB in acute infections and immune responses. The ability of FapB to fibrillate optimally in more acidic environments and FapC in neutral to slightly alkaline conditions may indicate spatial and temporal regulation within biofilms. FapB could initiate fibril formation in more acidic microenvironments, providing a scaffold during the early stages of biofilm development or in response to acute inflammation. In contrast, FapC could be more prominent in later biofilm maturation or less acidic conditions, contributing to biofilm integrity and resilience. This dynamic response ensures that the biofilm structure can adapt to fluctuating pH levels, providing a competitive advantage in diverse niches. Furthermore, the charge differences between FapB and FapC under various conditions might facilitate their interaction. For instance, between pH 5 and 6 in PAO1, FapB could be positively charged while FapC is negatively charged, suggesting potential charge–based interactions during oligomerization and fibrillation. These findings underscore the complexity of *Pseudomonas* biofilm regulation and highlight the importance of FapB and FapC in enabling these bacteria to thrive in diverse and challenging environments.

### Exceptional thermal and chemical resilience of FapB and FapC fibrils

The FapB and FapC fibrils demonstrate remarkable thermal stability, maintaining their fibrous structure even under severe conditions such as autoclaving. Moreover, autoclaving appears to enhance the incorporation of residual monomers into the fibril structure (Figs. 9 and S10). This resilience of amyloid fibrils to autoclaving, a process typically lethal to bacteria, has significant implications in both medical and industrial settings. These enduring proteins can adhere to medical instruments and various surfaces, potentially fostering environments favorable for the growth of new bacterial colonies. The presence of these residual amyloids from previous biofilms could facilitate the rapid colonization of surfaces that were initially bacteria-free. This phenomenon raises concerns about the effectiveness of autoclaving in completely eradicating biofilms, highlighting the necessity for a deeper understanding of how these remaining fibrils might promote subsequent bacterial colonization.

Chemically, Fap fibrils demonstrate remarkable stability, resisting disaggregation even under exposure to FA. Interestingly, when FapB is present, FapC fibrils exhibit enhanced resilience, with nearly half of the sample remaining in insoluble forms, even in the presence of concentrated, undiluted FA (Fig. 10). This is in stark contrast to the curli amyloids CsgA and CsgB, which show varying interaction dynamics; mixed fibrils of these proteins are less chemically stable than their individual counterparts (Fig. S11).

The heightened stability of Fap fibrils indicates their ability to persist under extreme conditions, which contributes to the long-term stability of biofilms. Such resilience suggests that standard sterilization and chemical treatments may be insufficient to eradicate these biofilm components, necessitating the development of more effective methods to disrupt and remove persistent biofilms. Moreover, numerous studies have demonstrated that microbial amyloids can cross-seed and influence the formation of human amyloids, potentially contributing to neurodegenerative diseases such as Alzheimer's and Parkinson’s (57, 58, 59, 60, 61, 62, 63). Notably, FapC has been shown to seed and accelerate the aggregation of Aβ fibrils in zebrafish models, even altering their cognitive functions (64). These findings underscore the urgent need to better understand and manage microbial amyloids to mitigate their potential impact on human health.

### Reevaluating the roles of FapB and FapC in *P. aeruginosa* PAO1

Bacterial functional amyloids, such as Fap proteins, undergo a highly regulated polymerization process, providing physiological benefits to bacteria (18). Nucleation, a pivotal aspect of this process, influences the formation, growth, kinetics, stability, and morphology of amyloid fibrils, along with their physiological and pathological role (13, 18, 19, 65). In *P. aeruginosa*, FapB has been hypothesized to initiate FapC fibrillation, similar to CsgB's role, considering the lower abundance of FapB in biofilms (4, 8, 11, 26, 40). However, our recent findings challenge this perspective.

When FapB and FapC from PAO1 are combined, we observed a prolonged lag phase in fibrillation (Fig. 3), indicating a delay in β-rich fibril formation. Additionally, SDS-PAGE analysis of remaining monomers revealed that in each other's presence, Fap proteins maintain higher solubility over 9 days than when isolated (Figs. 3 and 10). FapB's aggregation kinetics is more influenced by FapC than *vice versa*. Moreover, FapB fibril seeds did not significantly alter the fibrillation kinetics of FapC, whereas FapC seeds modestly enhanced FapB fibrillation (Fig. 4). Confocal microscopy supports this, showing that FapB aggregation predominantly colocalize with preexisting FapC aggregates, while the reverse is less common (Figure 5, Figure 6, Figure 7 and Videos S1 and S2). Both Faps showed slightly increased fibrillation with self-seeding (Fig. 4), aligning with patterns observed in other amyloids (66, 67, 68, 69).

AlphaFold3 modeled structures suggest that FapB and FapC can independently form characteristic Greek key β-solenoid structures, consistent with known amyloid cores that can interact to form single copolymer fibrils. However, the models indicate that as the number of protein units increases, there is a tendency for these proteins to form separate fibrils that bundle together rather than incorporate into single copolymer fibrils. This ability to form copolymer fibrils, linear fibrils, and bundled structures, as seen from the AlphaFold3 modeling, might suggest a versatile interaction mechanism that could be modulated depending on the environmental conditions and the specific ratios of FapB to FapC, supporting adaptive biofilm architecture.

Overall these findings suggest that FapB and FapC employ distinct nucleation and growth strategies, with their interaction in combined amyloid formations being complex and condition-dependent. This raises questions about the role of FapB as a primary nucleator for FapC, at least in the PAO1 orthologs, hinting at a more intricate relationship. The colocalization of Faps, facilitated by interactions between their monomers or fibrils, might lead to mixed fibrils or intertwined protofilaments, potentially enhancing the structural integrity of *P. aeruginosa* biofilms in diverse environments. Given FapC faster fibrillation and reduced pH dependency, it appears to be the primary amyloid for biofilm scaffolding, similar to CsgA. In contrast, FapB might serve as an auxiliary amyloid, fibrillating under specific conditions and integrating into the biofilm through self-nucleation or existing FapC fibrils. Moreover, the complex dynamics within the Fap system could be influenced by other proteins like FapA and FapE. FapA is known to modulate FapC fibrillation and alter FapB's distribution in mature fibrils (8, 9, 21, 40), while FapE acts as an extracellular chaperone during fibrillation and is present within mature amyloid fibrils (8, 25, 40). This intricate interplay of proteins within the Fap system exemplifies the sophisticated regulation of amyloid interactions in bacterial biofilms.

## Conclusions

Given the significant health and environmental impacts of *Pseudomonas* infections, particularly the resistance of *P. aeruginosa* biofilms to antimicrobial treatments and their ability to evade host defenses (70, 71, 72, 73, 74, 75), a deeper understanding of their virulent amyloids is essential. Our research uncovers the remarkable thermal and chemical resilience of FapB and FapC fibrils. This resilience indicates that biofilm structures may withstand standard sterilization processes, potentially contributing to the persistence of *P. aeruginosa* infections. The durability of these fibrils also presents exciting opportunities for developing robust bionanomaterials or protective coatings, capitalizing on their unique properties.

Our studies also illuminate the significant influence of environmental factors on Fap proteins, suggesting that homologs in different *Pseudomonas* strains may have adapted to specific environments and roles. Challenging the view of FapB as the primary nucleator, our findings indicate a more pronounced role of FapC in influencing FapB aggregation. However, the interplay between FapB and FapC is intricate: FapB aids in stabilizing FapC fibrils, while FapC can slow down the fibrillation of FapB, yet still serve as a cross-seeding template. This complex dynamic is the key to understanding their roles within bacterial biofilms. Furthermore, the observed distinctions between Fap and curli amyloids underscore the importance of comprehensively understanding different amyloid systems for the development of effective intervention strategies.

## Experimental procedures

### Materials and reagents

Competent *E. coli* BL-21 cells (New England Biolabs) were used for the transformation of pET-28b(+) plasmids (Novagen). PMSF protease inhibitor (Sigma-Aldrich); guanidinium HCl (SPECTRUM) were employed for the lysis buffer during protein purification. IPTG purchased from Ornat was used to induce protein expression. Cyanine3 NHS ester and Cyanine5 NHS ester fluorophore dyes were purchased from Lumiprobe and dissolved in dimethyl sulfoxide (Merck). A 15μ-Slide 8-well glass bottom/15μ-Slide VI 0.4 were purchased from Ibidi GmbH. HisPur cobalt resin beads (Thermo Fisher Scientific *via* Danyel Biotech) were used for protein purification. Luria-Bertani (LB) agar, Bacto Yeast, Bacto Tryptone (BD Biosciences), and Kanamycin antibiotic (MERCK group) were used for bacteria growing. GelRed Nucleic Acid Stain (Gold Biotechnology) was used for DNA gel staining. FA was purchased from Gadot Group. Ultrapure water (Biological Industries) were used throughout the study. ThT dye was purchased from Sigma-Aldrich. Acrylamide/bis-acrylamide 29:1 40% (BioLab), Ammonium Peroxydisulfate (Alfa Aesar) and N, N, N′, N′-tetramethylethylenediamine (Sigma-Aldrich) were used for gel preparation. SDS (Holland Moran group), DL DTT (Sigma-Aldrich) and ExcelBand 3-color Regular Range Protein Marker (SMOBIO) were used in SDS-PAGE analysis. InstantBlue Coomassie Protein Stain from Expedeon was used for protein gel staining. Four hundred mesh copper grids with support films Formvar/Carbon (Ted Pella Inc) and 1% uranyl acetate solution (Electron Microscopy Sciences Ltd) were used for negative staining in TEM. Thermal seal film (EXCEL scientific), and black 96-well flat-bottom plates (Greiner Bio-One) were used for kinetic assay measurements. Zeba spin desalting columns (Rhenium) and disposable polypropylene columns (Bio-Rad) were used for buffer exchange and purification steps. Potassium dihydrogen phosphate (Merck Group), Potassium phosphate dibasic (SPECTRUM), sodium acetate (Merck Group), Tris Base (Fisher Bioreagents), Bis-Tris hydrochloride (Sigma Aldrich), and sodium chloride (BioLab) were used for buffer preparation.

### Computational analyses and calculations of biophysical properties

To explore the biophysical attributes of FapB and FapC amyloids, we conducted a detailed computational analysis, focusing on their length, pH sensitivity, and charge characteristics. This investigation encompassed amyloid homologs from various *Pseudomonas* strains: *P. aeruginosa* PAO1 (UniProt# Q9I2E9, Q9I2F0), *P. fluorescens* Pf-5 (UniProt# Q4KC08, Q4KC07), *P. putida* F1 (UniProt# A5W4A4, A5W4A5), and *Pseudomonas* sp. UK4 (UniProt# C4IN69, C4IN70). We utilized the UniProt database and the Prot-pi web calculator (https://www.protpi.ch/Calculator/ProteinTool) for precise sequence analysis of each homolog, including length, charge, and isoelectric point. Prot-pi was also used to calculate the overall charge of each amyloid at different pH levels (4–9). For FapB and FapC from the *P. aeruginosa* PAO1 strain, used in our experiments, we calculated the overall charge at various pH levels also considering the impact of a histidine tag at the N- or C-terminal site.

Molecular insights into the protonation state of FapB under various pH conditions were obtained using the AlphaFold2 AI-based structure modeling tool (37, 38). Structure predictions for *P. aeruginosa* PAO1 FapB and its histidine-tagged variant were carried out on the online Google Colab service using AlphaFold v2.1.0 advanced. To assess the protonation effects on these structures, they were converted to PDB files and analyzed with the H++ automated system (76, 77). This system calculates pK values of ionizable groups in macromolecules and adds hydrogen atoms as needed for the specified environmental pH. The resulting structures, showing electrostatic potential, were visualized, colored, and analyzed using UCSF ChimeraX software (https://www.cgl.ucsf.edu/chimerax/) (78), allowing us to compare variations of charge distribution over the protein surface at different pH levels (Fig. S3).

To explore the molecular structure of FapB and FapC multimers, we utilized AlphaFold3, the latest version of the AI-driven tool that can predicts monomeric and multimeric protein structures (79). Models of *P. aeruginosa* PAO1 histidine-tagged FapB and FapC were generated at varying ratios in alignment with the experimental setup. All predictions were conducted using a constant seed value of 42, and only the top-ranked models were used. The resulting structures were visualized, colored, and analyzed using UCSF ChimeraX software (78).

The Clustal Omega Sequence Alignment tool (80, 81), was used to determine the sequence identity between FapB and FapC from *P. aeruginosa* PAO1, focusing on their full sequences, excluding signal peptides. Similarly, we analyzed the sequence identity between *E. coli* K12's CsgA and CsgB (UniProt# P28307, P0ABK7).

### Protein production of FapB and FapC

#### Construction of FapB and FapC plasmids

The genes of the PAO1 *P. aeruginosa* strain FapB and FapC (UniProt #: Q9I2E9 and Q9I2F0, respectively) were successfully cloned into the pET28b expression vector plasmid. We designed specific primers using the NEB site, ensuring they were complementary to targeted regions of the genes and included necessary restriction sites. These sites were strategically selected to allow simultaneous cutting of both the vector and the gene with the same restriction enzymes, thereby streamlining the gene insertion process.

The NEB Q5 High-Fidelity PCR Kit was utilized to amplify the genes from *P. aeruginosa* genomic DNA. Subsequently, the amplified genes were cloned into the pET28b(+) plasmid (Novagen). Notably, the pET28b vector incorporates a histidine tag at either the N- or C-terminal site, and the primers were tailored to align with specific segments of the gene sequence. This approach led to the creation of four distinct plasmids: two containing the FapB gene and two containing the FapC gene, each with a histidine tag positioned at one of the terminal sites.

To confirm the successful integration of the genes within the pET28b vector, several steps were undertaken. Initially, the plasmids were transformed into *E. coli* XL-1 competent cells and cultured on LB agar plates supplemented with 50 μg/ml kanamycin antibiotic. Following overnight incubation at 37 °C, selected colonies were propagated in LB medium. The plasmids were then extracted using the Presto Mini Plasmid Kit, and their concentrations were quantified with a NanoDrop spectrophotometer.

To ascertain the presence of the correct genes in the plasmids, we employed specific restriction enzymes (XhoI, NcoI, and NdeI from NEB) to cleave the plasmids at predetermined sites corresponding to the gene insertion. The resultant plasmid fragments were separated *via* electrophoresis on a 1% agarose DNA gel, which was stained with GelRed Nucleic Acid Stain. The gel documentation was performed using the Bio-Rad Gel imaging system to verify the precise insertion of the genes into each plasmid. Finally, to authenticate the gene sequences, DNA sequencing was conducted by Macrogen-Syntezza Bioscience Ltd, ensuring the accuracy and integrity of our plasmid constructs.

The sequences are as follows:

#### FapC

MGNEGGWHPPKPNPQSNNKGGATALVVDTQQNYNNKVSNFGTLNNASVSGSIKDASGNVGVNVAAGDNNQQANAAALASADASFVFGTATASTSVLQSGYGNTLNNYSNPNTASLSNSANNVSGNLGVNVAAGNFNQQKNDLAAAVSNGQYSTAGSAASQTSTGNTTVNSANYAYGGTYVSLKLNADGSYKGTSDQIGDVYLDTWEGQTHPGGSNTGHIDVDSQAQGAKDLNHDGGAFAFKEKGDVDLKGTVSGFIPAIVGFKTPVTNNASLSNSLQNVSGNVGVNIAAGGGNQQSNSLSIAAGCSSCPAGGESLGFLEHHHHHH.

#### FapB

MGSSHHHHHHSSGLVPRGSHMDPLASRNRASIDDSGTYRGNFALNQAAGDAQQQSNVRVVAVGSAALVGSDQRQHLQLDASQPIAASASISGAALRGSGILGVNQGAGLGNQQINAFRLSLSNGPESLDDSVLAQSVALTKVSGSATPVPGGRSVSTDDRAFAGSSGVVQVNQSAGVGNQSMNTLSVRVME.

#### Expression and purification of FapB and FapC

The production of FapB and FapC proteins was carried out separately using the following protocol. All buffers were filtered through a 0.22 μm Millex filter and maintained at 4 ˚C. The purified pET28b plasmids, containing either the FapB or FapC genes with a histidine tag at the N- or C-terminal site, were transformed into competent *E. coli* BL-21 cells. These were then plated on LB agar plates supplemented with 50 μg/ml kanamycin antibiotic and incubated overnight at 37 °C. Colonies from these plates were transferred to 100 ml LB medium containing the antibiotic and grown overnight at 37 °C.

The overnight cultures were diluted into 700 ml of the same medium and incubated at 37 °C with shaking at 220 rpm until an absorbance (*A*) of 0.8 to 0.9 at 600 nm was reached. Protein expression was induced by adding 1 mM IPTG, followed by a 3-h incubation under the same conditions. The bacterial cells were harvested by centrifugation at 4500 rpm for 30 min and stored at −80 °C.

##### Protein purification

For purification, HisPur cobalt resin beads (Thermo Fisher Scientific) were used. The cell pellets were thawed, resuspended in 30 ml of lysis buffer (6 M guanidinium HCl, 50 mM potassium phosphate, 0.1 mM PMSF, pH 7.4), and incubated overnight at 4 °C with agitation. The lysate was centrifuged at 17,000*g* for 30 min at 4 °C to separate into supernatant and pellet fractions. The supernatant was incubated with 2 ml of equilibrated HisPur cobalt resin beads in lysis buffer for 2 to 3 h at 4 °C with agitation. The mixture was then loaded onto a disposable polypropylene column at 4 °C and washed with 20 ml of 50 mM potassium phosphate buffer (pH 7.4), followed by a wash with the same buffer containing 10 mM imidazole. Proteins were eluted using 350 mM imidazole in 50 mM potassium phosphate buffer (pH 7.4). Imidazole was removed, and the buffer was exchanged using Zeba spin desalting columns (7 k MWCO, Thermo Fisher Scientific) at 4 °C into different buffers as required for specific experiments.

##### Protein concentration and verification

Protein concentrations were determined by measuring absorbance at 280 nm using extinction coefficients of 24,535 M^−1^ cm^−1^ for FapC and 1490 M^−1^ cm^−1^ for FapB, calculated using the Expasy server. The identity of FapB and FapC proteins was confirmed through Western blot analysis using an anti-histidine tag antibody.

### Purification and labeling of FapB and FapC with fluorophores

To observe the formation of FapB and FapC fibrils, we utilized two fluorophore dyes: Cyanine3 NHS ester (Excitation: 555 nm, Emission: 570 nm) and Cyanine5 NHS ester (Excitation: 646 nm, Emission: 662 nm) from Lumiprobe. These NHS ester dyes covalently bond with primary amines, predominantly lysine, in proteins, ensuring stable labeling. Each fluorophore was dissolved in dimethyl sulfoxide to achieve a concentration of 4 mg/ml. Protein lysate samples were split into two portions. One served as an unlabeled control, while the other was designated for labeling.

#### Labeling process

The supernatant fraction was mixed with 1 ml of HisPur cobalt resin beads pre-equilibrated with lysis buffer and agitated for 1 to 2 h at 4 °C. After loading the mixture onto a disposable polypropylene column, it was washed with 10 ml of 50 mM potassium phosphate buffer, followed by a wash with the same buffer containing 10 mM imidazole. A 2:1 M ratio of dye to protein was prepared for either Cyanine3 or Cyanine5 NHS ester, using the concentration of the unlabeled fraction as a reference. The dye solution was then mixed with the HisPur-bound amyloid samples (FapB with Cyanine3 and FapC with Cyanine5) and incubated on the column for 2 h at 4 °C in the dark. Additional washes were performed, including a quenching buffer (50 mM Tris–HCl, pH 7.4), followed by two washes with 50 mM potassium phosphate buffer. The labeled amyloids were eluted, and imidazole was removed as previously described. The protein aliquots were stored at −80 °C.

#### Concentration determination and verification

To quantify the fluorophore-tagged FapB and FapC, SDS-PAGE analysis was conducted. Tagged and untagged protein samples were heated at 95 °C for 10 min with a sample buffer containing SDS and DTT, then loaded onto 15% polyacrylamide gels and run at 120 V for 60 min. A Typhoon FLA 70000 Phosphorimager was used to confirm correct labeling, followed by staining with InstantBlue Coomassie Protein Stain for 30 min. Gel imaging was performed using a Bio-Rad gel doc system, and the concentrations of the fluorophore-dyed proteins were determined by comparing band intensities to those of the known concentrations of the untagged proteins.

### Fluorescence microscopy analysis of FapB and FapC

To investigate colocalization in time-lapse imaging, labeled FapB and FapC protein samples were first thawed. Depending on the experimental setup, they were either mixed in a 1:1 ratio to reach a concentration of 50 μM in 50 mM potassium phosphate buffer and subjected to 24 h of time-lapse imaging or incubated separately at 37 °C in the dark for 24 h before being mixed at the same ratio and then subjected to 6 h of time-lapse imaging. Both the mixed samples and the unmixed controls were placed onto 8-well chamber slides (15μ-slides 8-well glass bottom, Ibidi) for observation under a confocal microscope. Imaging was performed using a Nikon Ti2-Eclipse microscope, equipped with a 100×/1.35 silicone immersion objective (Nikon CFI SR HP Plan Apochromat Lambda S), along with 561 nm and 640 nm laser diodes. Photometrics BSI sCMOS cameras were used to capture the images, focusing on the bottom of the well to observe residing aggregates. Time-lapse imaging was conducted at 37 °C. Additionally, to investigate the colocalization of FapB and FapC monomers with preformed fibrils, samples were prepared by mixing FapB monomers (0 h incubation) with preformed FapC fibrils (72 h incubation) and FapC monomers (0 h incubation) with preformed FapB fibrils (72 h incubation). Fluorescence images were captured at two-time points: immediately after mixing (0 h) and after 24 h of incubation. These mixed samples were then placed onto microscope slides with cover glasses for observation under a confocal microscope. Imaging in this experiment was performed using a Leica DMI8 microscope at 20× magnification. In all experimental settings, fluorescence images were acquired with filters specific for Cyanine5 and Cyanine3. All confocal microscopy experiments were repeated twice to ensure reproducibility.

#### Colocalization analysis

Time-lapse images were recorded and subsequently corrected for photobleaching using the Napari plugin with a biexponential method. Fluorescence imaging data analysis was performed using Imaris 10.1 software (Oxford Instruments; https://imaris.oxinst.com/versions/10-1). For each experiment, a fixed intensity threshold was applied to ensure uniform comparison between the images. Co-localization was evaluated by creating composite images that combined the Cyanine5 and Cyanine3 channels, with yellow/orange regions indicating areas of intersection between FapB and FapC fibrils.

### ThT fluorescence kinetic assays for monitoring fibrillation of FapB and FapC

ThT assays are a standard method for studying amyloid fibril formation. We used this approach to investigate the fibrillation kinetics of FapB and FapC under various conditions, including different pH levels, ionic strengths, coincubation ratios, and the presence of preformed fibril seeds.

#### ThT assay of Faps at varied pH and ionic strength conditions

Freshly purified monomers of FapB and FapC were transitioned from the elution buffer to the desired condition buffer using Zeba spin desalting columns. Based on preliminary assessments, we selected N-terminal tagged FapB for its higher fibrillation propensity (Fig. S3) and C-terminal tagged FapC, as the tag's position showed no significant impact on its fibrillation. Initial fibrillation tests were conducted in a 50 mM potassium phosphate buffer at pH 7.4. To explore the effect of pH, we used a "universal buffer," a solution that maintains a stable pH across a wide range, from acidic to basic, making it suitable for diverse biochemical and biophysical experiments. It provides consistent buffering capacity, reduces interactions with proteins and metal ions, and remains stable at different temperatures. Here, we worked with a "universal buffer" consisting of 20 μM Tris, 20 μM Bis-Tris, and 20 μM sodium acetate covering a pH range of 3.5 to 9.2 (41). ThT assays were performed by combining FapB or FapC monomers at concentrations of 25, 50, and 100 μM with 20 μM ThT prepared as a stock solution in ultrapure water and diluted in the respective pH buffer, in a total volume of 100 μl. As controls, wells containing only 20 μM ThT were included for each pH value examined. Additionally, to assess the influence of ionic strength on fibrillation, we used a pH 7 buffer supplemented with NaCl to achieve final concentrations of 0, 0.15, and 0.3 M NaCl. The reaction mixtures were placed in black 96-well flat-bottom plates (Greiner Bio-One) covered with thermal seal film (EXCEL scientific), and incubated in a plate reader (FLUOstar Omega, BMG Labtech) at 37 °C, with orbital shaking at 300 rpm for 30 s prior to each measurement. ThT fluorescence was recorded every 3 min, utilizing an excitation wavelength of 438 ± 20 nm and an emission wavelength of 490 ± 20 nm. All measurements were performed in triplicate, and the entire experiment was repeated at least three times.

#### ThT assays of Faps at different coincubation ratios

To investigate the impact of interaction/nucleation between FapB and FapC monomers on their fibrillation kinetics, we mixed freshly purified FapB monomers with increasing concentrations of FapC monomers in the "universal buffer" at pH 5, along with filtered ThT prepared in ultrapure water. The final concentrations in the reaction wells were 50 μM FapB mixed with 0, 2, 5, 10, 25, 50, 75, or 100 μM FapC, along with 20 μM ThT, in a final volume of 100 μl. Control wells contained only FapC at each concentration and ThT, replacing FapB with an equivalent volume of the "universal buffer" at pH 5. A reciprocal experiment was conducted by mixing FapC monomers with increasing concentrations of FapB monomers at pH 7, following the same protocol with the same concentrations. In this case, the control wells exclusively contained FapB and ThT at pH 7. The measurement settings were consistent with those described in the previous paragraph. The measurements themselves were performed in triplicate, and the entire experiment was repeated at least three times.

#### ThT assays of self-seeding and cross-seeding of the Faps

The interaction between FapB and FapC was further investigated by assessing the influence of preformed sonicated fibrils, referred to as "seeds," on the fibrillation kinetics of their respective monomers using a ThT kinetic assay. For this purpose, we incubated 50 μM of FapB or FapC with varying volumes of preformed sonicated fibrils of FapB and FapC. To produce FapB and FapC fibrils, freshly purified monomers were incubated in a final volume of 350 μl and a concentration of 100 μM in a "universal buffer" at pH 5 or 7 (respectively) for 1 week at 37 °C with shaking at 300 rpm. The fibrils were then dried under a vacuum for 24 h, followed by resuspension in their original buffer and vigorous shaking. Subsequently, the fibrils were bath sonicated at room temperature for 5 min. For further sonication, the fibrils were placed on ice and subjected to VC750 VibraCell (Sonics) tip sonication at 30% amplitude, with three 20-s bursts at 50-s intervals.

To perform the measurements, freshly purified FapB monomers were mixed with increasing volumes of preformed sonicated fibrils of FapB and FapC (self-seeding and cross-seeding) in the same buffer at pH 5. Similarly, FapC monomers were mixed with the corresponding preformed sonicated fibrils of FapB and FapC, with the only difference being the pH set to 7. The final concentrations in the reaction wells were 50 μM FapB or FapC monomers mixed with 0, 2, 5, and 10% (v/v) of seeds, along with 20 μM ThT, in a final volume of 100 μl. Controls included wells containing only the seeds at each concentration, along with ThT and the respective pH. The measurement settings remained consistent with those described in the previous paragraph. All measurements were performed in triplicate, and the entire experiment was repeated at least three times.

### Transmission electron microscopy

TEM was utilized to visualize the fibrils of FapB and FapC, providing insights into the effects of various fibrillation conditions and treatments on their structural formation. Samples for TEM analysis were collected either at the conclusion of the ThT kinetic assays or at predetermined intervals based on the assay's protocol. In instances where the fibrils underwent specific treatments, such as temperature variations or exposure to different concentrations of FA, samples were taken directly from these treated conditions.

For TEM grid preparation, a volume of 5 μl from the sample was applied onto 400 mesh copper grids. These grids were equipped with support films made of Formvar/Carbon, obtained from Ted Pella, and were charged using high-voltage, alternating current glow-discharge immediately prior to sample application. This preparation ensured optimal adherence of the samples to the grids. After allowing the samples to settle on the grids for 2 min, they were subjected to negative staining using a 1% uranyl acetate solution for an additional 2 min.

The imaging of the samples was conducted using an FEI Tecnai G2 T20 S-Twin transmission electron microscope. This microscope operated at an accelerating voltage of 200 KeV, enabling high-resolution visualization of the fibril structures. The micrographs were captured at the MIKA Electron Microscopy Center, which is part of the Department of Material Science and Engineering at the Technion–Israel Institute of Technology.

### SDS-PAGE analysis of FapB and FapC residual monomers

To assess the stability of FapB and FapC fibrils, we conducted SDS-PAGE analysis to quantify residual monomers, considering that insoluble fibrils do not migrate in SDS-PAGE even after boiling. This characteristic is shared with other amyloids involved in biofilm formation, such as CsgA (44). The distinct molecular weights of FapC (∼32.6 kDa) and FapB (∼19.4 kDa) allowed clear separation of the proteins on the gel. All raw gels are provided in Figs. S12–S16.

#### SDS-PAGE analysis of FapB and FapC self-seeding and cross-seeding

FapB and FapC monomers were prepared at a concentration of 50 μM and incubated with 10% v/v preformed seeds, either from themselves or their counterpart protein. The incubation took place in a universal buffer, with pH 5 for the FapB monomer sample and pH 7 for the FapC monomer sample. Samples were collected at 0, 48, and 72 h and were then prepared for SDS-PAGE analysis following the protocol described in the subsequent section. The intensity of the monomer bands at each time point was quantified, normalized to the 0 h sample, and expressed as a percentage, with the values displayed above each band. The entire experiment was replicated twice, showing a similar trend across independent experiments. The raw gels for this experiment are provided in the Fig. S13.

#### SDS-PAGE analysis of FapB and FapC thermostability

We investigated the thermostability of FapB and FapC fibrils, as well as mixed fibrils formed by combining both amyloids. Freshly purified FapB and FapC were incubated separately in a universal buffer at pH 5 and 7, respectively. For mixed fibril samples, we prepared two sets, one at pH 5 and the other at pH 7. Each amyloid was at a final concentration of 50 μM, and in the mixed samples, they were combined in a 1:1 ratio. These samples were incubated for 4 days at 37 °C with shaking at 300 rpm. Following this initial incubation, the samples underwent thermal treatments. One set was incubated at 85 °C for 0, 6, and 24 h, while another set was exposed to extreme temperatures by autoclaving at 121 °C. Posttreatment, each sample was mixed with SDS sample buffer supplemented with DTT and heated at 95 °C for 10 min. To quantify the amount of monomers obtained posttreatment, we collected a sample of fresh monomers before the initial incubation and fibril formation as a control.

The samples were then loaded onto a 15% acrylamide SDS-PAGE gel and subjected to electrophoresis at a constant voltage of 120 V for 60 min. After electrophoresis, we used Coomassie Stain (Expedeon-InstantBlue Coomassie Protein Stain) to visualize and stain the soluble monomers of FapB and FapC migrating within the gel. Gel imaging and quantification were performed using a Gel Doc–Bio-Rad system. The quantified values reported were calculated by normalizing the intensity of the posttreatment bands to that of a control band, of the untreated monomer sample of the same quantity. This normalization ensures that all numerical values displayed in the graphs represent the percentage of monomers remaining in the samples relative to the initial monomer quantity. Statistical analysis was conducted to evaluate significant differences in monomer levels between treatment groups. We applied a one-way ANOVA to compare monomer levels among groups (incubated and incubated + autoclaved) and determine whether any group means significantly differed. Tukey’s honest significant difference *post hoc* test was used to identify specific pairwise differences. Results were reported with *p* values, where significance levels are indicated as *p* < 0.01 (∗∗) and *p* < 0.001 (∗∗∗). The entire experiment was replicated twice for the heat treatment and a minimum of three times for the autoclaving process. The quantification of the results from the autoclaving treatment was derived from data across three separate, independent experiments. The raw gels for these experiments are provided in the Figs. S14 and S15.

#### SDS-PAGE analysis of FapB and FapC stability in FA

To assess the chemical stability of FapB and FapC amyloid fibrils, we focused on their interaction with FA, a solvent known for its ability to disrupt amyloid structures, including dissolving amyloid beta plaques (46, 47). In this experiment, we prepared samples of FapB and FapC separated or mixed, following the methodology outlined in the previous section. However, in this instance, we allowed the samples to undergo fibrillation for an extended period of 7 to 10 days.

Postfibrillation, the samples were dried using a SpeedVac (RVC 2–18 CDplus, CHRIST) to eliminate excess liquid and concentrate the fibrils. Subsequently, each dried amyloid sample was reconstituted in varying concentrations of FA, diluted in ultrapure water. The FA concentrations ranged from 0% to 100%, with increments of 25%. We then adjusted the volume of each resuspended sample back to its original 200 μl volume prior to drying. Following resuspension, the samples were incubated for 10 min at 25 °C and then rapidly frozen using liquid nitrogen. Overnight lyophilization (ScanVac Cool Safe-Freeze dryers, LaboGene) was employed to remove any remaining liquid from the samples. For the analysis, the lyophilized amyloids were reconstituted to their initial volume and concentration of 50 μM using the appropriate "universal buffer" at either pH 5 or pH 7. The reconstituted samples were then subjected to SDS-PAGE, normalized, and analyzed as previously described. Statistical analysis was conducted to evaluate significant differences in monomer levels across treatment groups. A one-way ANOVA was applied to compare monomer levels among groups treated with different FA concentrations (0%, 25%, 50%, 75%, and 100%) to assess the effects of FA on the stability of FapB and FapC fibrils. Tukey’s honest significant difference *post hoc* test was used to identify specific pairwise differences. Results are presented with *p*-values, with significance levels indicated as *p* < 0.05 (∗) and *p* < 0.01 (∗∗). The entire experiment was replicated three times, and quantification and statistics were derived from data across three separate, independent experiments. The raw gels for this experiment are provided in the Fig. S12.

### SDS-PAGE analysis of *E. coli* CsgA and CsgB stability in FA

#### *E. coli* CsgA and CsgB protein production

Electro-competent BL21-DE3 slyD KO cells were electroporated with pET11a plasmids carrying histidine-tagged CsgA or CsgB genes. These transformed cells were cultured on LB-agar plates with ampicillin at 37 °C. A single colony was used to initiate a preculture, which then started the main expression culture in 2xYT medium supplemented with ampicillin and glucose. This culture was incubated at 37 °C with shaking until reaching a specific bacterial density, followed by IPTG induction and further incubation. Postincubation, the culture was centrifuged, and the cell pellet was stored or prepared for purification.

*Purification*: The cell pellet was dissolved in 7.5 M guanidine hydrochloride, centrifuged to remove debris, and then incubated with nickel-nitrilotriacetic acid beads (Qiagen) for binding. After several washes and elution steps, the protein concentration was determined using a DeNovix DS-11 Nanodrop. The purified protein was filtered, snap-frozen, and stored. For assays, the protein was thawed, desalted, and its concentration reassessed.

#### Fibrillation of CsgA and CsgB

Fibrils were generated from protein samples with concentrations of 30 μM. These samples were incubated at 37 °C in Eppendorf tubes, either individually or mixed in a 1:1 M ratio, for 48 h. Postincubation, the fibrils underwent a triple washing process with centrifugation at 13,000*g* for 10 min. The supernatant was removed, and the fibril pellet was resuspended in fresh 50 mM Tris buffer (pH 7.4). The protein concentration in the supernatant was quantified. The washed fibrils were sonicated using a Fisherbrand 505 Sonicator.

#### FA stability assay

The sonicated fibril samples were transferred to new Eppendorf tubes and centrifuged at 13,000*g* for 10 min. The supernatant was discarded, and the fibril pellet was treated with 50 μl of X% FA, mixed, and vortexed gently. After a 20-min room temperature incubation, the samples were centrifuged again. A portion of the supernatant was flash-frozen in liquid nitrogen, lyophilized, and reconstituted in 2× Laemmli SDS-PAGE loading buffer. The samples were then loaded onto a Bio-Rad TGX Stain-Free 4 to 20% SDS-PAGE gel and electrophoresed.

Protein bands were detected using Coomassie staining and stain-free imaging. While Coomassie staining could not differentiate between CsgA and CsgB, Stain-free imaging selectively visualized CsgA based on its tryptophan residues. Band intensities were analyzed using Bio-Rad’s Image Lab Software (https://www.bio-rad.com/en-dk/product/image-lab-software?ID=KRE6P5E8Z) and normalized against those observed in the 98% FA sample for comparative analysis. The raw gels for this experiment are provided in the Fig. S16.

## Data availability

All raw data generated in this study are either provided in the article and supplementary information (*e.g.*, raw SDS-PAGE gels) or available upon request from the corresponding author.

## Supporting information

This article contains supporting information.

## Conflict of interest

The authors declare that they have no conflicts of interest with the contents of this article.
