# Supplementary material for: Resilience and charge-dependent fibrillation of functional amyloid: Interactions of Pseudomonas biofilm-associated FapB and FapC amyloids
Source: J Biol Chem. 2024 Dec 18;301(2):108096. doi: 10.1016/j.jbc.2024.108096 (PMC11787515; doi:10.1016/j.jbc.2024.108096)
Supplement: Supporting Information [file mmc1.pdf]

Supplemental Information

Resilience and Charge-Dependent Fibrillation: Interactions of *Pseudomonas* Biofilm-Associated FapB and FapC Amyloids

Nimrod Golan, Amit Parizat, Orly Tabachnikov, Eilon Barnea, William P. Olsen, Daniel E. Otzen and Meytal Landau*

* To whom correspondence should be addressed: meytal.landau@desy.de (M.L)

The supplemental information contain:
Table S1
Figures S1-S16

Table S1. Comparison of length, pI, charge, and molecular weight between four FapB and FapC orthologs

	Pseudomonas	*Length*	*Isoelectric point*	*Charge at pH 7*	*Molecular Weight [kDa]*
FapB	*sp. UK4 (C4IN69)*	164	8.5	0.94	16.8
	fluorescens Pf-5 (Q4KC08)	174	8.5	0.84	17.5
	putida F1 (A5W4A4)	174	5.2	-1.64	17.9
	aeruginosa PAO1 (Q9I2E9)	170	5.8	-1.04	17.1
	aeruginosa PAO1 His N-ter	191	7.4	0.64	19.4
	aeruginosa PAO1 His C-ter	178	6.7	-1.07	18.1
FapC	*sp. UK4 (C4IN70)*	226	6.9	-0.13	22.6
	fluorescens Pf-5 (Q4KC07)	305	5.7	-5.59	30.7
	putida F1 (A5W4A5)	459	5.1	-14.02	46.6
	aeruginosa PAO1 (Q9I2F0)	316	4.9	-6.91	31.5
	aeruginosa PAO1 His N-ter	336	6.1	-5.16	33.7
	aeruginosa PAO1 His C-ter	324	5.7	-6.94	32.6

Sequence properties were calculated using the Prot-pi: Protein Tool for Isoelectric Point and Net Charge Calculation. Protein Analysis Website: https://www.protpi.ch/

Figure S1. Impact of Histidine tag position on FapC fibrillation kinetics.

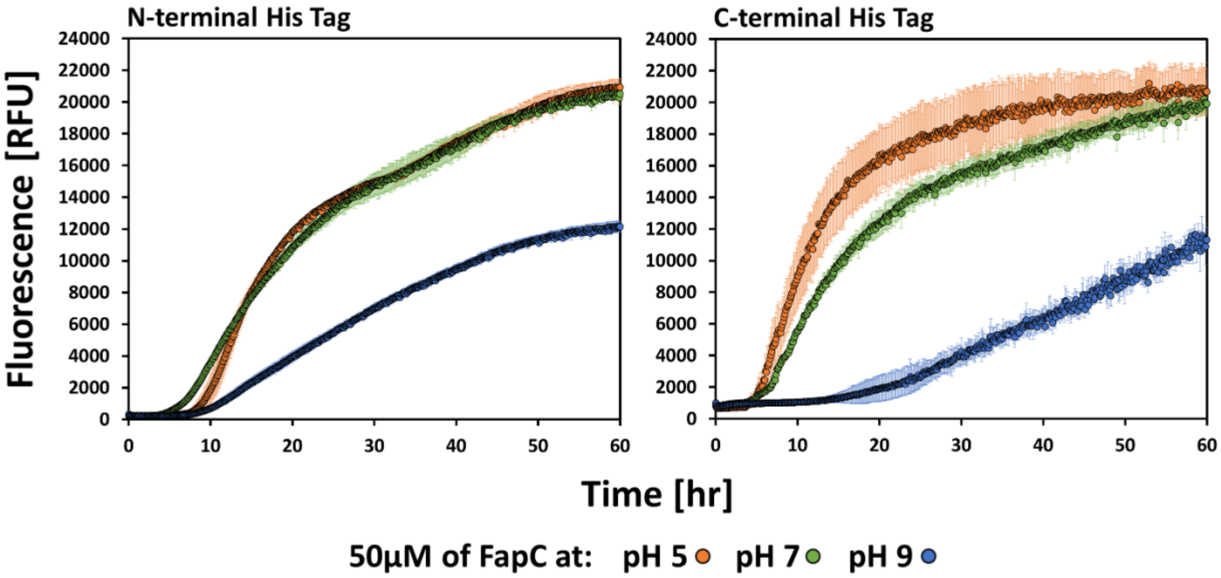

ThT fluorescence data showing the fibrillation of 50 µM FapC with Histidine tags at the N-terminus (left panel) and C-terminus (right panel) across three pH conditions: pH 5 (orange), pH 7 (green), and pH 9 (blue). The fibrillation kinetics were monitored over 60 hours. Error bars represent the standard deviation from the mean of triplicate measurements. These results demonstrate that both N-terminal and C-terminal histidine tags do not significantly influence the fibrillation of FapC, with similar results and variations observed across different pH levels.

Figure S2. Impact of Histidine tag position on FapB fibrillation kinetics and net charge

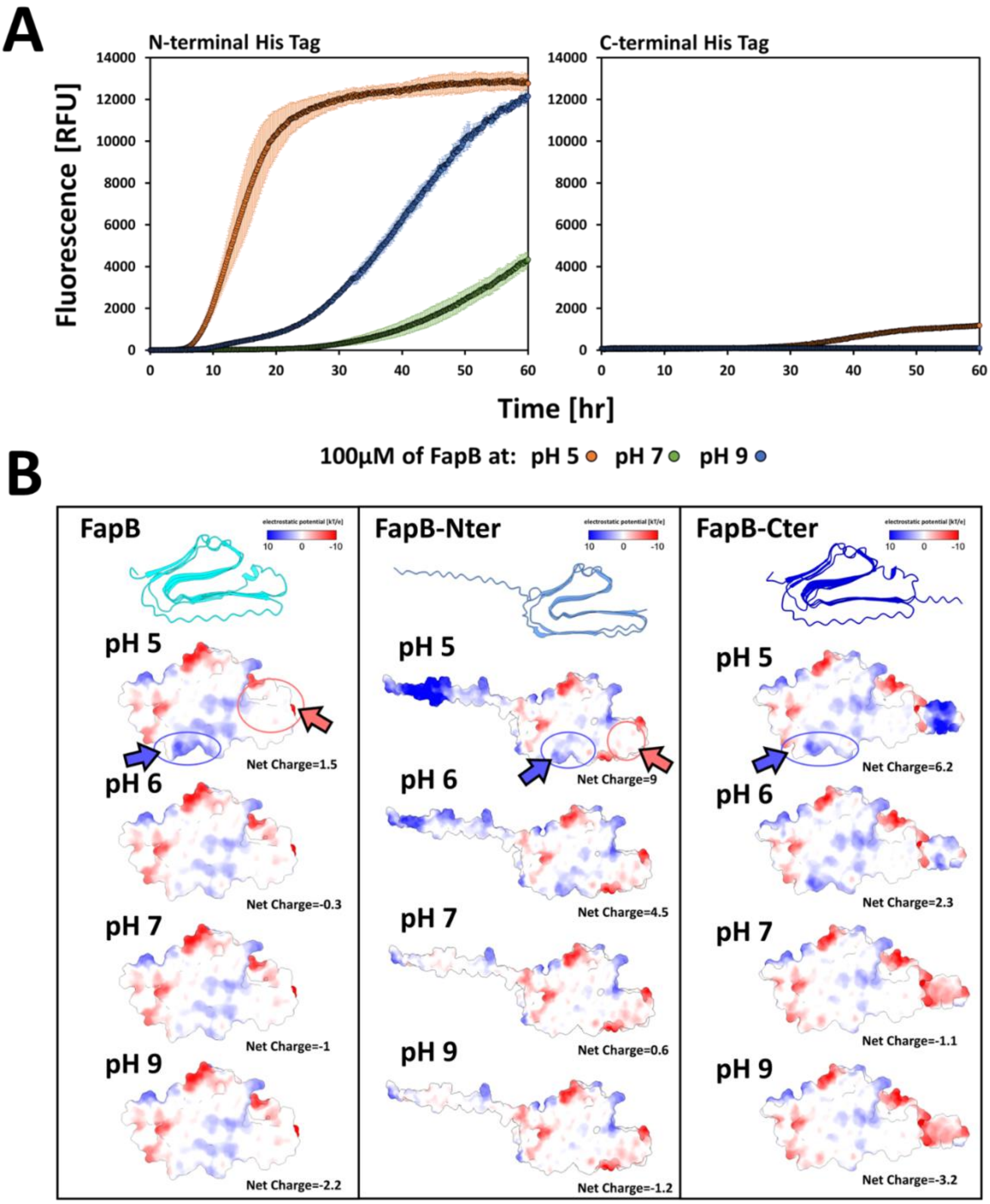

(A) ThT fluorescence data showing the fibrillation of 100 μM FapB with Histidine tags at the N-terminus (left panel) and C-terminus (right panel) across three pH conditions: pH 5 (orange), pH 7 (green), and pH 9 (blue). The fibrillation kinetics were monitored over 60 hours. Error bars represent the standard deviation from the mean of triplicate measurements. (B) Surface charge distribution of modeled structures of FapB (Q4KC08) predicted by AlphaFold, both

with and without Histidine tags at the N- and C-terminal sites. FapB was predicted to form a folded monomer adopting a Greek-key β-solenoid structure. Charge distribution, calculated with the H++ web tool under four pH conditions, is presented on the surface of the models shown in the same orientation. Negative and positive charges are colored red and blue, respectively, with a charge scale provided. Significant charge changes are marked with arrows and circles. Additionally, the total charge is displayed at each pH level.

Figure S3. Influence of ionic strength on fibrillation kinetics of Histidine-tagged FapB and FapC

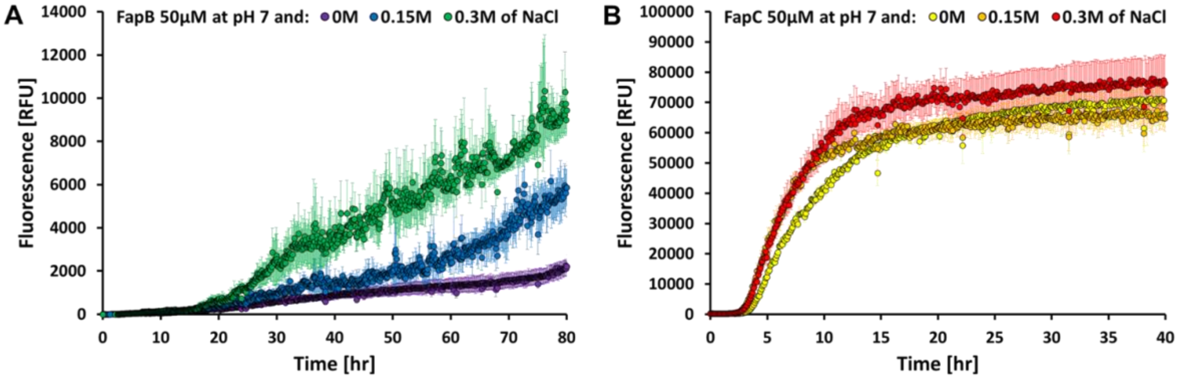

This figure presents ThT fluorescence data reflecting the fibrillation of 50 µM FapB (A) and FapC (B) at pH 7 under three different NaCl concentrations (0, 0.15, and 0.3M). Error bars in the graphs represent the standard deviation from the mean of triplicate measurements. The experiment was replicated on three separate occasions, consistently showing similar trends.

Figure S4. Effect of pH on fibrillation rates of Histidine-tagged FapB and FapC at various concentrations

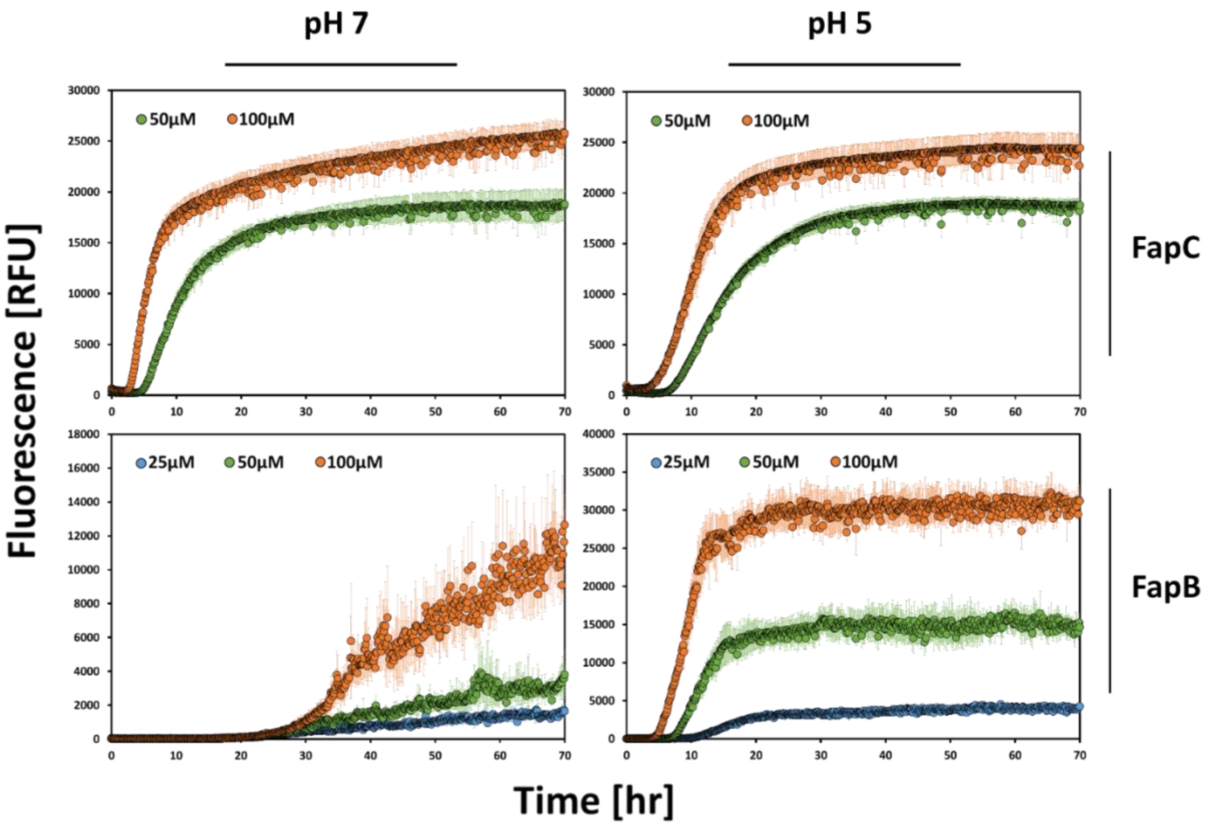

This figure displays ThT fluorescence data demonstrating the fibrillation rates of 50 and 100 µM FapC at pH 7, and of 25, 50, and 100 µM FapB at pH 5. The error bars on the graphs represent the standard deviation from the mean of triplicate measurements. These experiments were replicated on three different days, consistently producing similar results.

Figure S5. Extended analysis of fibrillation kinetics in co-incubated FapB and FapC monomers and control samples at various ratios and pH conditions

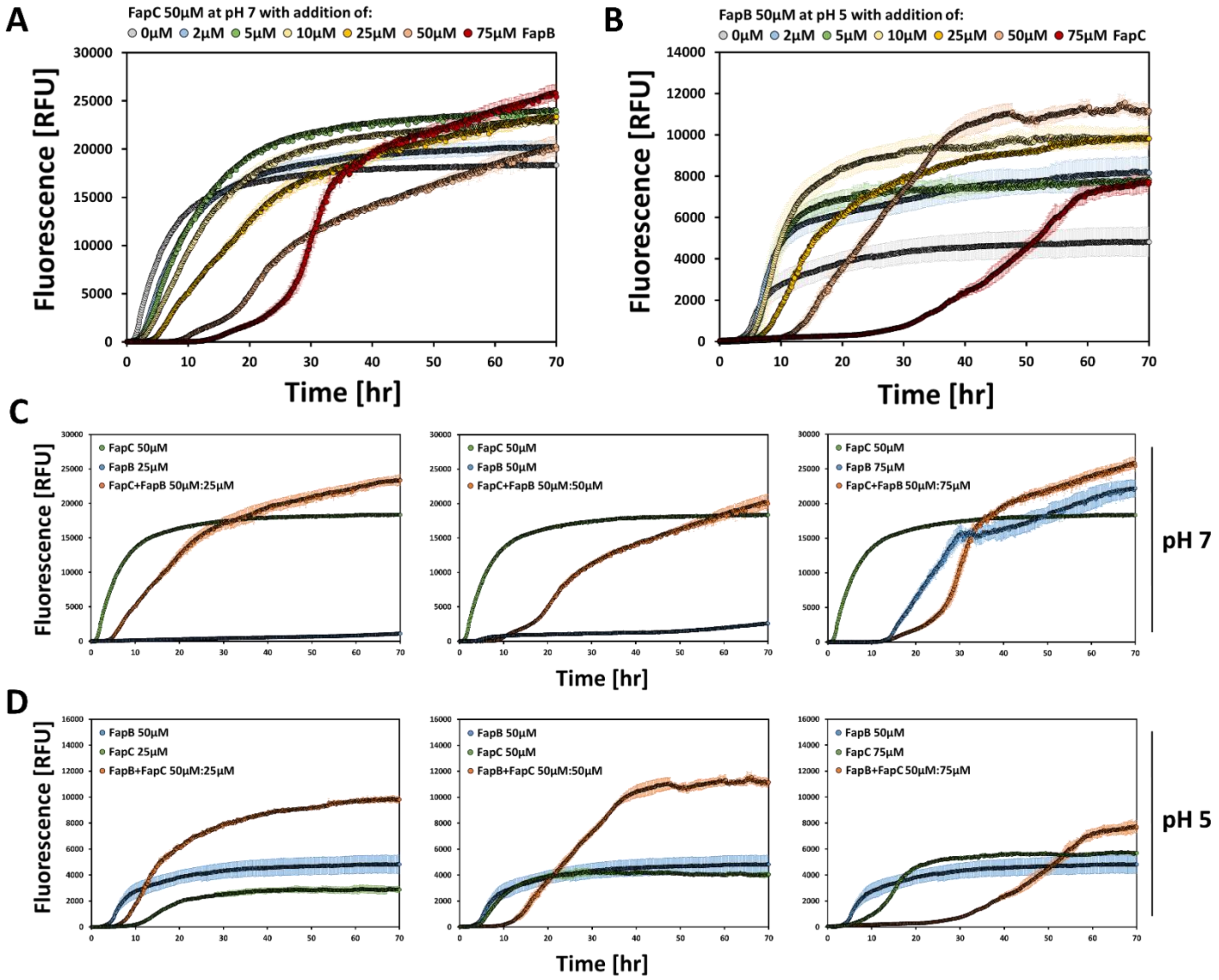

(A-B) ThT fluorescence kinetics depict the fibrillation rates of 50 µM FapC monomers at pH 7 (A) and FapB monomers at pH 5 (B), co-incubated with varying concentrations of their counterpart monomers (0 to 75 µM). Panels C-D showcase control experiments, presenting ThT measurements of fibrillation rates for each Fap monomer at both pH 7 (C) and pH 5 (D), both in isolation and when mixed. This section serves as an expanded version of Figure 3A-B, which focused on a 50 µM 1:1 Fap molar ratio (reproduced here in the central panels), and here includes additional concentrations and molar ratios. Error bars on the graphs represent the standard deviation from the mean of triplicate experiments, which were consistently replicated on different days, yielding similar trends.

Figure S6. The fluorophore labeling of FapB and FapC does not hinder their ability to form fibrils

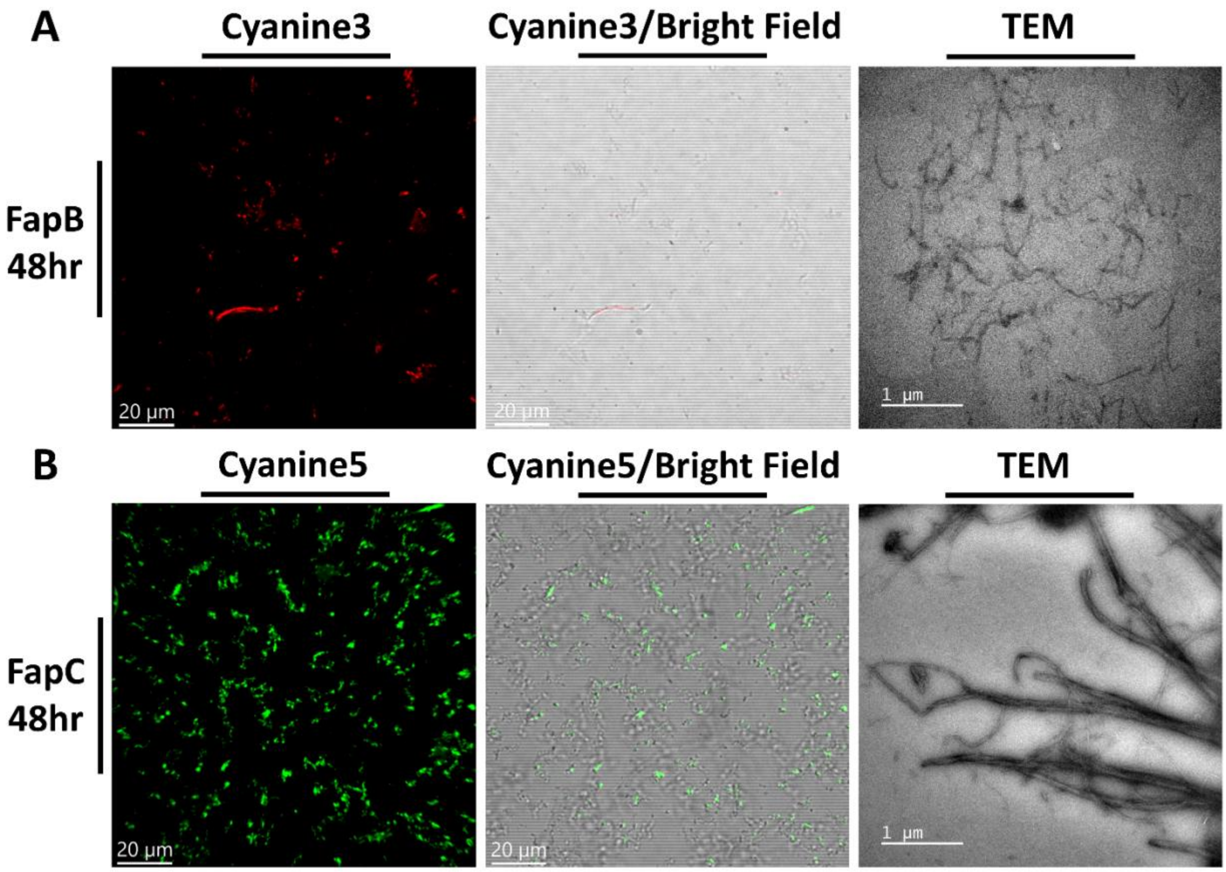

Fluorescence microscopy and TEM images of 50 μM FapB and FapC after 48 hours of incubation. FapB is labeled with Cyanine3 (visualized in red), and FapC is labeled with Cyanine5 (visualized in green). (A) FapB samples are shown in the fluorescence channel (left), combined with the bright field (BF) channel (middle), and visualized using Transmission Electron Microscopy (TEM) (right). (B) FapC samples are presented in the fluorescence channel (left), combined with the BF channel (middle), and imaged using TEM (right). TEM images show elongated fibrils for both FapB and FapC, indicating that labeling does not prevent fibril formation. Scale bars represent 20 μm for fluorescence and bright field images and 1 μm for TEM images.

Figure S7. Control for photobleaching influence in confocal microscopy co-localization experiments

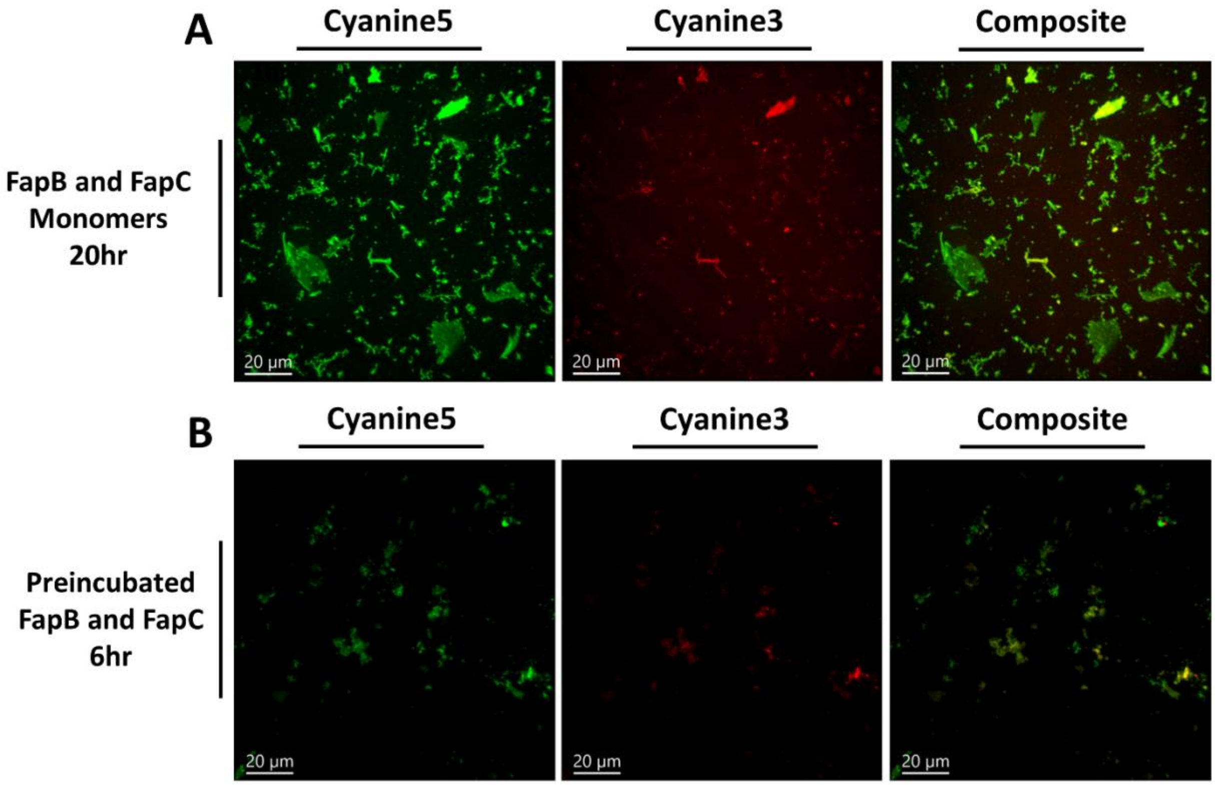

(A) FapB and FapC monomers co-incubated for 20 hours, with FapC labeled with Cyanine5 (visualized in green) and FapB labeled with Cyanine3 (visualized in red). This sample serves as a duplicate control from the same batch presented in Figure 5, but it is visualized only at the endpoint. The composite image (right) shows the overlap of the two channels, highlighting areas of co-localization. (B) FapB and FapC were pre-incubated separately for 24 hours, then mixed and incubated for an additional 6 hours. The labeling is the same, with Cyanine5 (green) for FapC and Cyanine3 (red) for FapB. The composite images highlight areas of co-localization. Scale bars represent 20 µm. This figure serves as a control to ensure that observed fluorescence patterns are not due to photobleaching effects, demonstrating the co-localization of FapB and FapC under different incubation conditions.

Figure S8. Impact of heat treatment on residual monomers of FapB and FapC

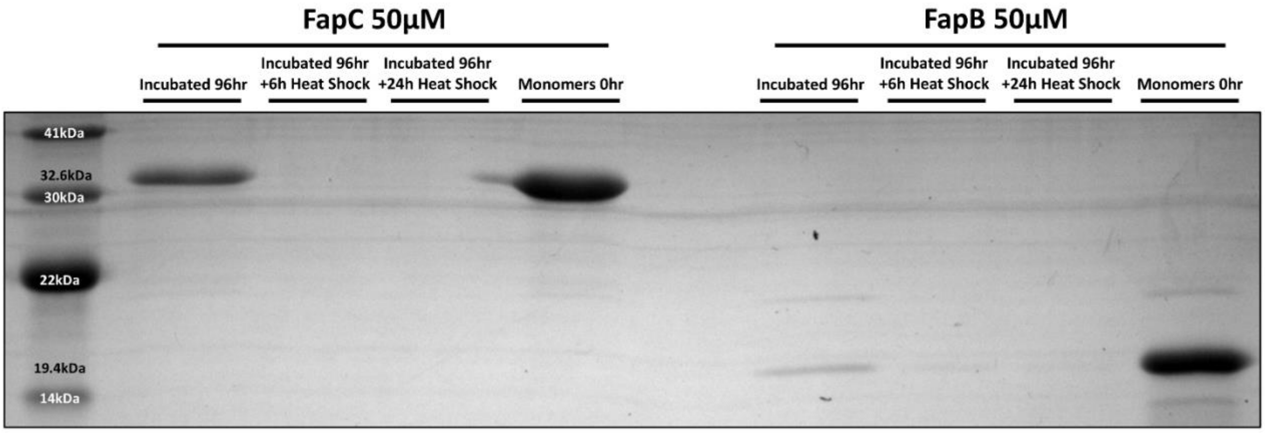

SDS-PAGE analysis of 50 µM FapB or FapC, incubated for 96 hours at pH 5 and 7, respectively, and then subjected to heat treatment at 85°C for 6 hours and 24 hours, with comparisons to fresh monomers (0 hours), and 96-hour incubated samples. A molecular weight marker is included in the gel to provide precise size references, simplifying the distinction between protein bands. The distinct molecular weights of FapC (~32.6 kDa) and FapB (~19.4 kDa) allow for clear identification of the proteins. The experiment was repeated twice on different days, yielding similar trends.

Figure S9. FapB and FapC fibrillation observations over time and following heat treatment visualized by fluorescence microscopy.

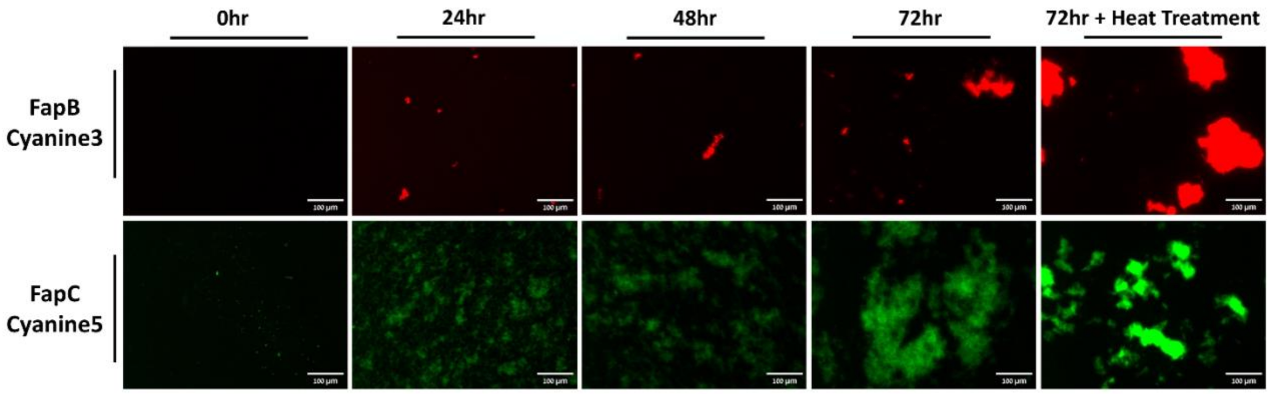

Fluorescence microscopy images showing the fibrillation of FapB and FapC over time. FapB is labeled with Cyanine3 (visualized in red), and FapC is labeled with Cyanine5 (visualized in green). Samples were taken at different time points: 0 hours, 24 hours, 48 hours, and 72 hours. An additional sample was taken after 72 hours of incubation, followed by heat treatment of 24hr incubation at 85°C. Top row: FapB samples showing increasing fibril formation over time, with significant aggregation observed after 72 hours and further enhancement following heat treatment. Bottom row: FapC samples displaying a gradual increase in fibril formation over time, with noticeable aggregation at 72 hours and increased fluorescence after heat treatment. Scale bars represent 100 µm.

Figure S10. Impact of autoclaving on residual monomers of FapB and FapC

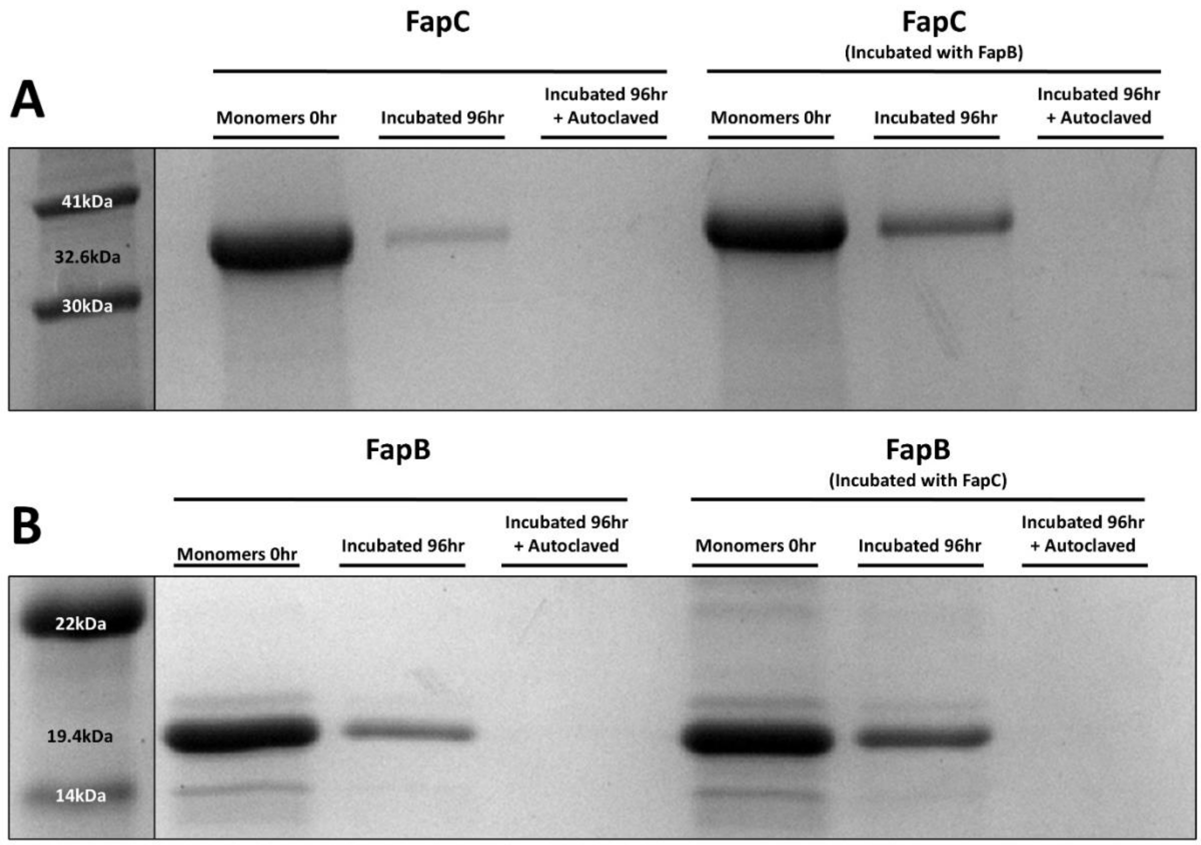

SDS-PAGE analysis of 50 µM FapC at pH 7 (A) and FapB at pH 5 (B), each incubated for 96 hours either separately or together with 50 µM of their counterpart. The analysis compares these samples before and after being autoclaved at 121°C for 20 minutes alongside fresh monomers (0 hours) and 96-hour incubated samples. Molecular weight markers are included adjacent to each gel to provide precise size references, fsimplifying the distinction between protein bands.

The distinct molecular weights of FapC (~32.6 kDa) and FapB (~19.4 kDa) allow for clear identification of the proteins. The experiment was repeated at least three times on different days, yielding similar trends. Quantification and statistical values from repeated experiments are presented in Figure 9.

Figure S11. The stability and interplay of CsgA and CsgB fibrils in the presence of formic acid

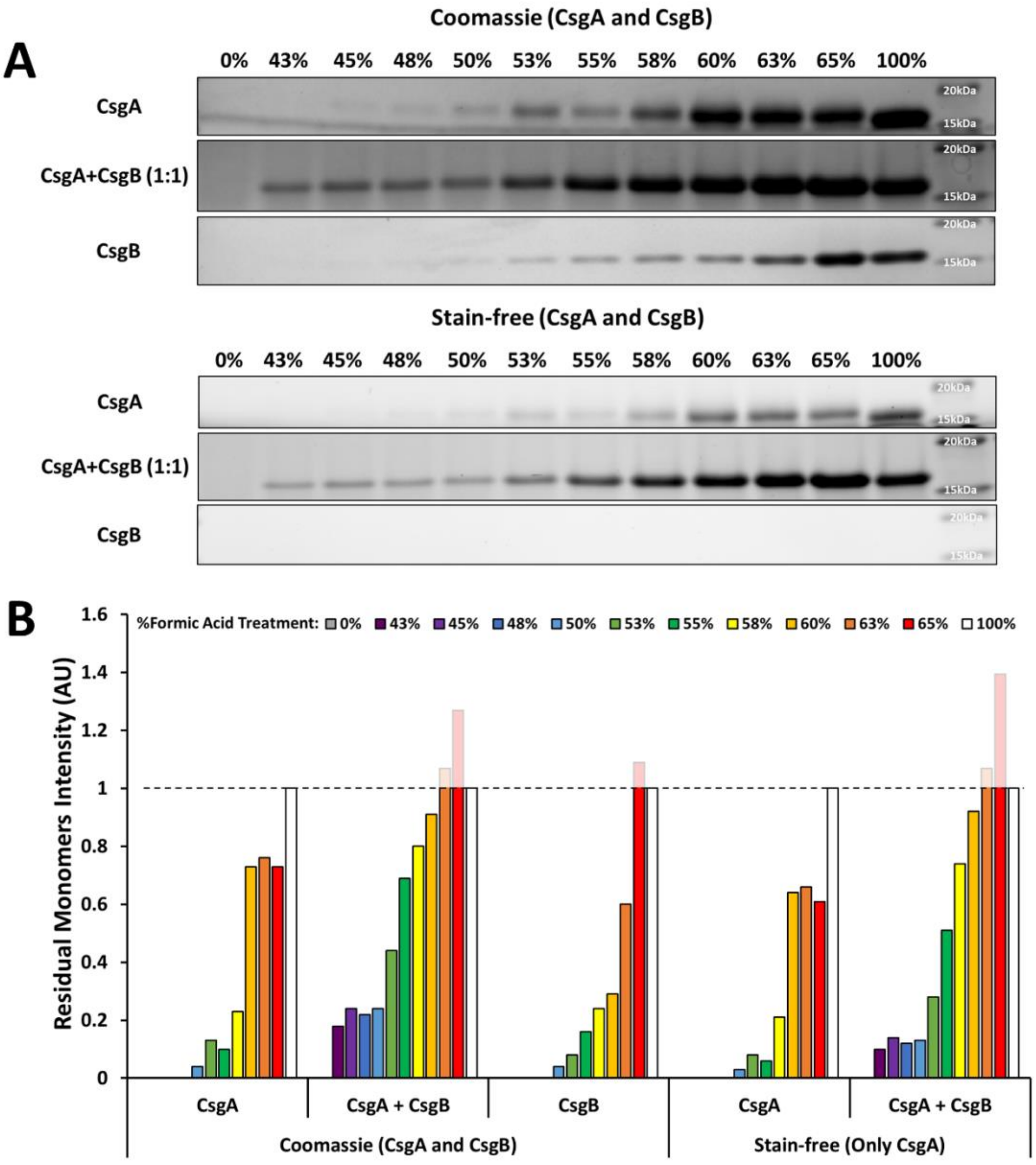

(A) A comparative SDS-PAGE analysis of CsgA and CsgB incubated either separately or in a mixture at a 1:1 molar ratio for 48 hours in a 50 mM Tris buffer with a pH of 7.4. Post-incubation, the CsgA and CsgB fibrils were subjected to various concentrations of formic acid. Molecular weight markers are indicated. Band detection was conducted using two distinct methods: Coomassie staining, while effective, cannot differentiate between CsgA and CsgB due to their similar molecular weights. In contrast, Stain-free imaging, which specifically detects tryptophan residues, exclusively identifies CsgA, as CsgB is devoid of tryptophan. (B)

A quantitative analysis of the SDS-PAGE band intensities, which represent residual monomers. The intensities are normalized against the baseline established under 100% formic acid (FA) treatment, marked by a dashed line. Notably, in the mixed CsgA/B samples, it appears that monomer disaggregation is almost complete at 63% FA concentration, although some experimental variations are observed at higher FA percentages.

Figure S12. Full-length SDS-PAGE gels corresponding to Figures 3 and 10

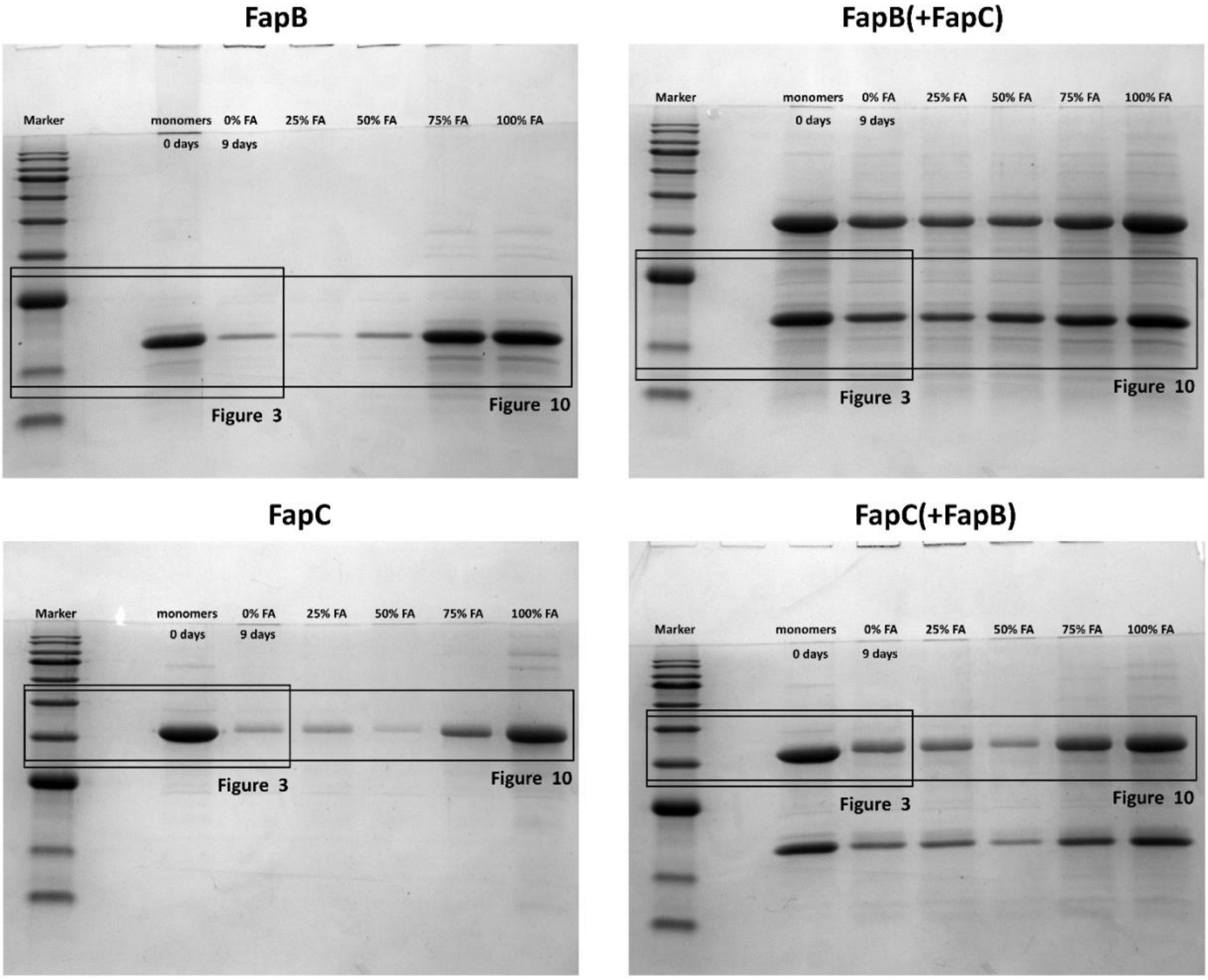

Full SDS-PAGE gels displaying the banding patterns of FapB, FapC, and their mixtures as presented in Figures 3 and 10. The gels represent samples of 50 µM FapB and FapC incubated individually or together at a 1:1 molar ratio for 0 days and 9 days, followed by treatment with varying concentrations of formic acid (FA): 0%, 25%, 50%, 75%, and 100%. The boxed areas indicate the specific sections shown in Figures 3 and 10.

Figure S13. Full-length SDS-PAGE gels corresponding to Figure 4

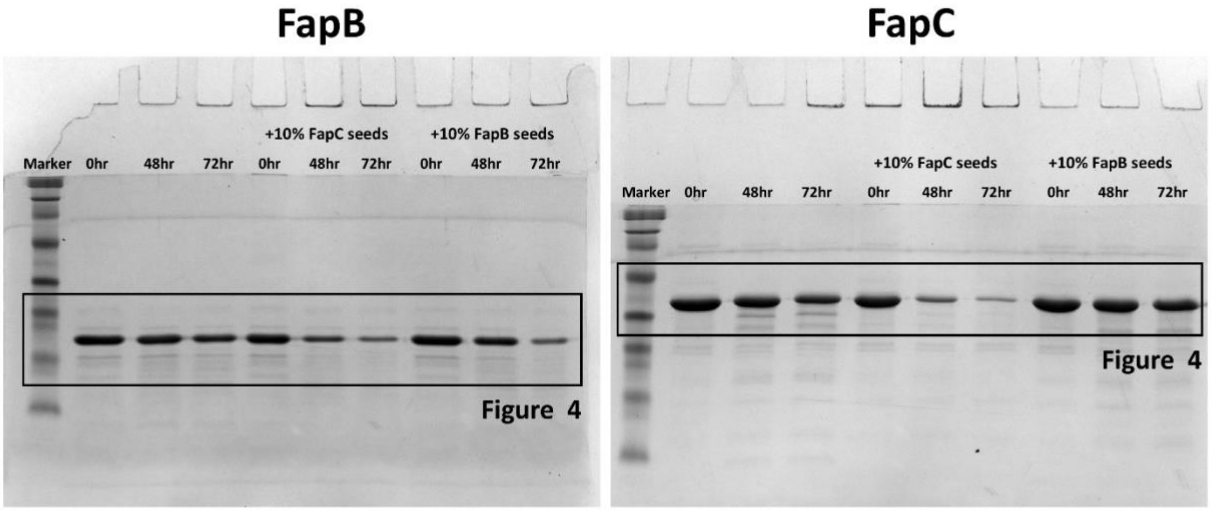

Full SDS-PAGE gels displaying the banding patterns of FapB and FapC, analyzed with 10% seeding by either FapB or FapC, as described in Figure 4. The gels represent samples of 50 µM FapB and FapC collected at time points of 0 hours, 48 hours, and 72 hours. The boxed areas indicate the specific sections presented in Figure 4.

Figure S14. Full-length SDS-PAGE gels corresponding to Figure S8

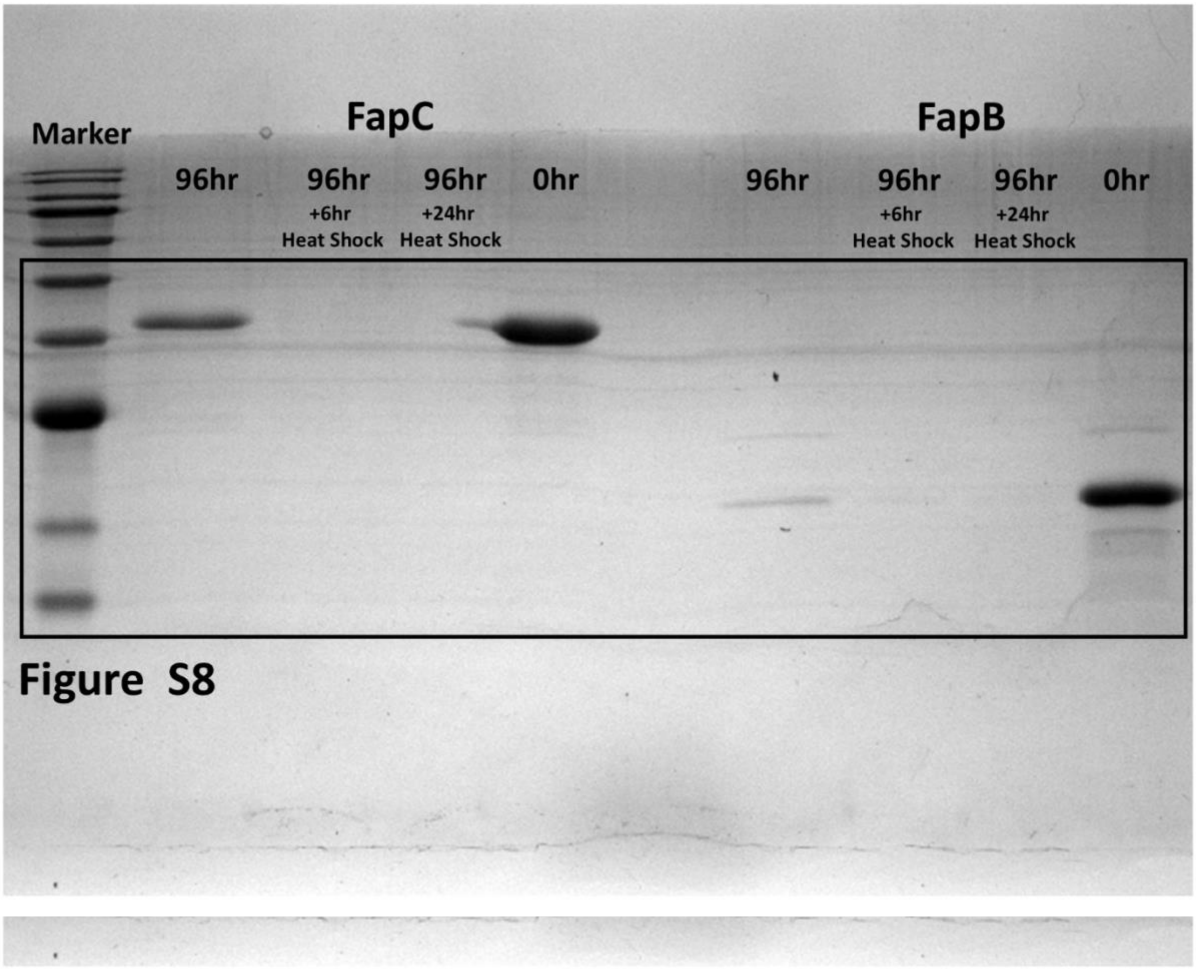

Full SDS-PAGE gel displaying the banding patterns of FapB and FapC as shown in Figure S8. This gel represents samples of 50 µM FapB and FapC after 96 hours of incubation, with and without heat shock treatment. The boxed area highlights the specific bands presented in Figure S8.

Figure S15. Full-length SDS-PAGE gels corresponding to Figure S10

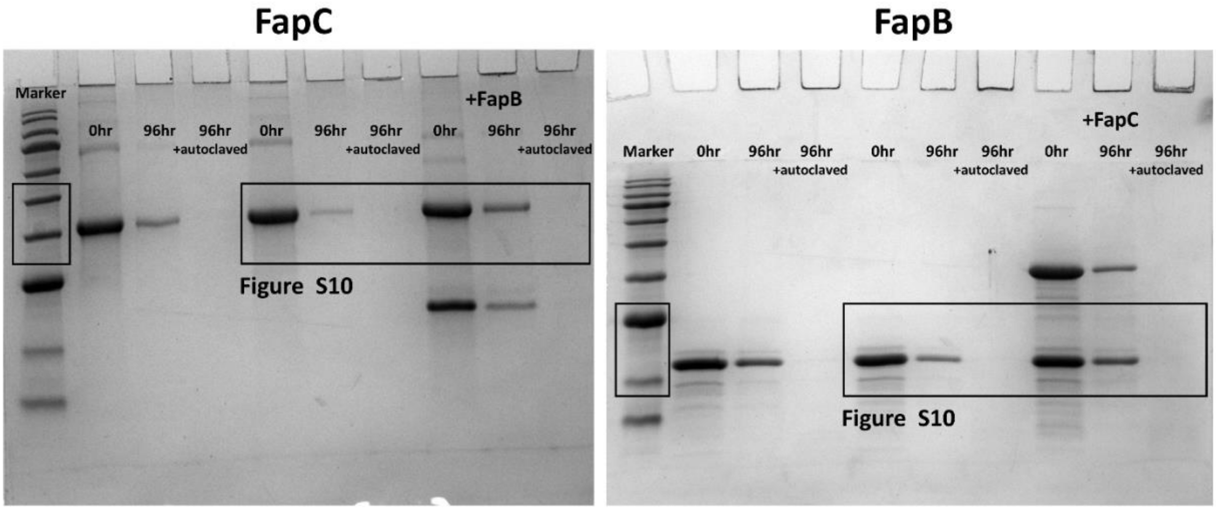

Full SDS-PAGE gels displaying the banding patterns of FapB and FapC as shown in Figure S10. These gels represent samples of 50 µM FapB and FapC after 96 hours of incubation, with and without autoclaving, including additional lanes for FapB and FapC mixtures. The boxed areas highlight the specific bands presented in Figure S10.

Figure S16. Full-length SDS-PAGE gels corresponding to Figure S11

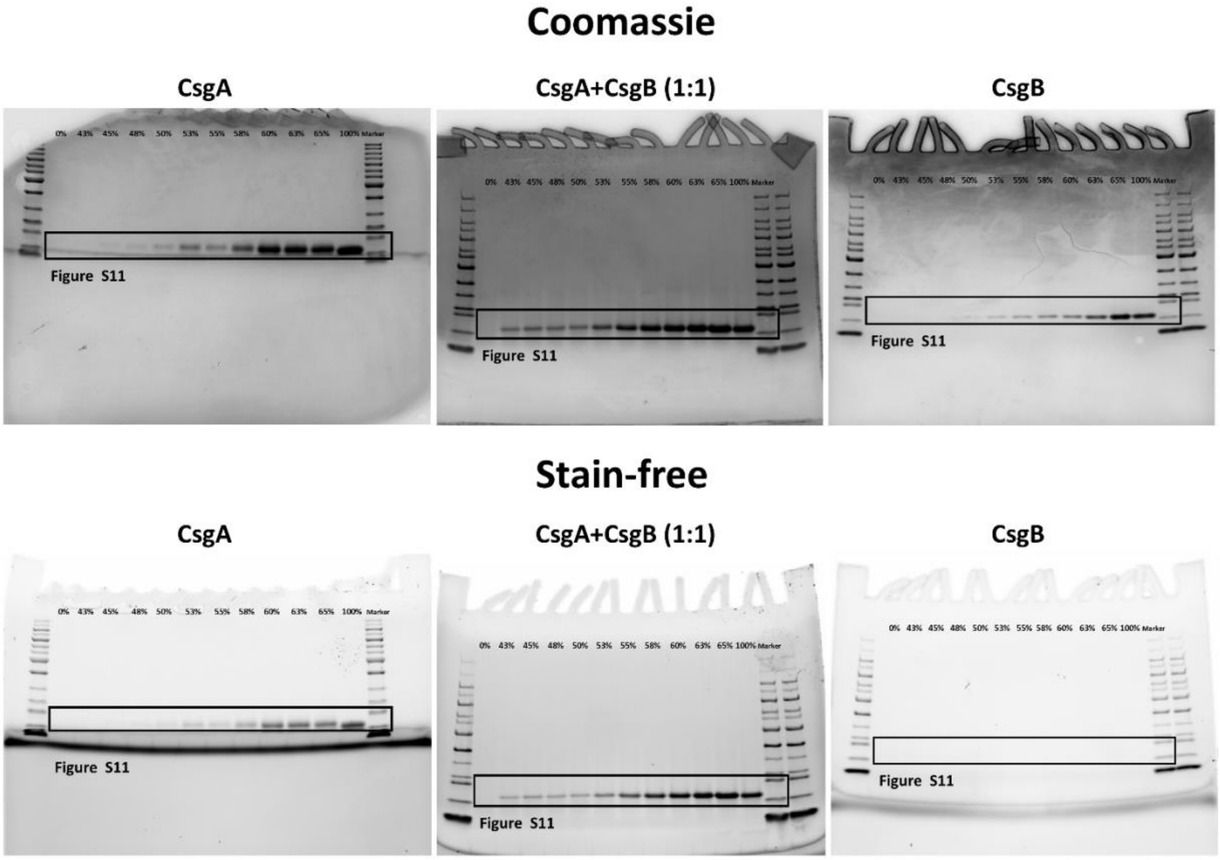

Full SDS-PAGE gels displaying the banding patterns of CsgA and CsgB, either individually or in a 1:1 mixture, as shown in Figure S11. The top row shows Coomassie-stained gels, which visualize the total protein content of both CsgA and CsgB. The bottom row shows Stain-free imaging gels, which selectively highlight CsgA due to its tryptophan content, as CsgB lacks tryptophan residues. The boxed areas indicate the specific bands presented in Figure S11.
